# Multiunit Frontal Eye Field Activity Codes the Visuomotor Transformation, But Not Gaze Prediction or Retrospective Target Memory, in a Delayed Saccade Task

**DOI:** 10.1523/ENEURO.0413-23.2024

**Published:** 2024-08-06

**Authors:** Serah Seo, Vishal Bharmauria, Adrian Schütz, Xiaogang Yan, Hongying Wang, J. Douglas Crawford

**Affiliations:** ^1^Centre for Vision Research and Centre for Integrative and Applied Neuroscience, York University, Toronto, Ontario M3J 1P3, Canada; ^2^Department of Neurosurgery and Brain Repair, Morsani College of Medicine, University of South Florida, Tampa, Florida 33606; ^3^Department of Neurophysics, Philipps-Universität Marburg, 35032 Marburg, Germany; ^4^Center for Mind, Brain, and Behavior – CMBB, Philipps-Universität Marburg, 35032 Marburg, and Justus-Liebig-Universität Giessen, Giessen, Germany; ^5^Departments of Psychology, Biology, Kinesiology & Health Sciences, York University, Toronto, Ontario M3J 1P3, Canada

**Keywords:** eye movements, frontal cortex, macaques, multiunit, single-unit

## Abstract

Single-unit (SU) activity—action potentials isolated from one neuron—has traditionally been employed to relate neuronal activity to behavior. However, recent investigations have shown that multiunit (MU) activity—ensemble neural activity recorded within the vicinity of one microelectrode—may also contain accurate estimations of task-related neural population dynamics. Here, using an established model-fitting approach, we compared the spatial codes of SU response fields with corresponding MU response fields recorded from the frontal eye fields (FEFs) in head-unrestrained monkeys (*Macaca mulatta*) during a memory-guided saccade task. Overall, both SU and MU populations showed a simple visuomotor transformation: the visual response coded target-in-eye coordinates, transitioning progressively during the delay toward a future gaze-in-eye code in the saccade motor response. However, the SU population showed additional secondary codes, including a predictive gaze code in the visual response and retention of a target code in the motor response. Further, when SUs were separated into regular/fast spiking neurons, these cell types showed different spatial code progressions during the late delay period, only converging toward gaze coding during the final saccade motor response. Finally, reconstructing MU populations (by summing SU data within the same sites) failed to replicate either the SU or MU pattern. These results confirm the theoretical and practical potential of MU activity recordings as a biomarker for fundamental sensorimotor transformations (e.g., target-to-gaze coding in the oculomotor system), while also highlighting the importance of SU activity for coding more subtle (e.g., predictive/memory) aspects of sensorimotor behavior.

## Graphical abstract

**Figure:**
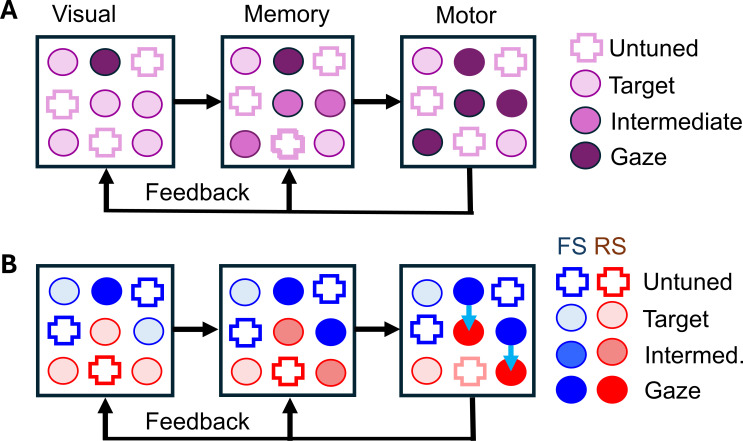
Visual Abstract

## Significance Statement

Multiunit recordings (undifferentiated signals from several neurons) are relatively easy to record and provide a simplified estimate of neural dynamics, but it is not clear which single-unit signals are retained, amplified, or lost. Here, we compared single-/multiunit activity from a well-defined structure (the frontal eye fields) and behavior (memory-delay saccade task), tracking their spatial codes through time. The progressive transformation from target-to-gaze coding observed in single-unit activity was retained in multiunit activity, but other cognitive signals (gaze prediction within the initial visual response, target memory within the final motor response, and cell-specific delay signals) were lost. This suggests that multiunit activity provides an excellent biomarker for healthy sensorimotor transformations, at the cost of missing more subtle cognitive signals.

## Introduction

In systems neuroscience, it is common to estimate neuronal population dynamics by relating recordings of neuronal activity to some concurrent task or behavior. Traditionally, neuroscientists have isolated single-unit (SU) action potentials of individual neurons from raw recorded background noise, based on their unique spike shape, a process called spike sorting. Recently, however, there has been increased interest in the use of multiunit (MU) activity (i.e., unsorted signals recorded from the tip of an electrode, which may arise from several nearby neurons (Buzsáki, 2004; Quian Quiroga and Panzeri, 2009; Panzeri et al., 2015; Rossi-Pool and Romo, 2019; Ayar et al., 2023). It has been suggested that MU activity recordings have practical and theoretical implications for experimental and applied electrophysiology (Fraser et al., 2009; Choi et al., 2010; Mattia et al., 2010; Smith et al., 2013; Sharma et al., 2015; Trautmann et al., 2019; Ahmadi et al., 2021; Leuthardt et al., 2021; Zhang and Constandinou, 2021). However, it remains unclear which signals are retained, and which are lost, when MU activity is left undifferentiated. Our aim was to test this question in a well-defined sensorimotor system (the frontal eye fields, FEFs), task (memory-delay saccades), and analytic technique, as described below.

SU recordings remain important and necessary for many applications. For example, it is necessary to first discriminate SU activity before it can be sorted into different cell types based on their sensory, motor, or biophysical properties (Ding and Gold, 2012; Vinck et al., 2013; Bharmauria et al., 2016b; Lowe and Schall, 2018; Trainito et al., 2019; Schneider et al., 2023). However, spike sorting poses both practical and theoretical challenges. The detection and extraction of SU among a sea of extracellular signals is often hindered by variability in spike shape/amplitude and extraneous background noise (Lewicki, 1998; Mtetwa and Smith, 2006). Isolation of single action potentials is further confounded by overlapping spikes, making it hard to identify and classify spikes (Quiroga et al., 2004; Mtetwa and Smith, 2006; Quian Quiroga and Panzeri, 2009; Rossi-Pool and Romo, 2019). These are time-consuming procedures that normally can only be done after recordings are complete. Further, neurons do not work in isolation but as ensembles forming functional networks (Hebb, 1949; Edelman, 1987; Buzsáki, 2010; Singer, 2013; Molotchnikoff et al., 2019; Levy et al., 2020).

MU activity analysis sidesteps these challenges by avoiding the need to identify unique spike waveforms. The process of extracting/sorting MU activity is more reproducible since it does not eliminate data or depend on specific sorting techniques/parameters. Some studies found that MU activity provided a more stable signal with higher spatial resolution than SU activity (Lewicki, 1998; Xing et al., 2009; Sharma et al., 2015; Drebitz et al., 2019; Ayar et al., 2023). Further, MU activity profiles are similar to local field potential (LFP) signals, but their activity tends to show less correlation with the activity of nearby neurons (Burns et al., 2010; Drebitz et al., 2019; Telenczuk and Destexhe, 2020; Ahmadi et al., 2021). Finally, MU analysis is capable of decoding the task and behavior by reducing the dimensionality of neural states (Quian Quiroga and Panzeri, 2009; Smith et al., 2013; Rossi-Pool and Romo, 2019; Trautmann et al., 2019; Ayar et al., 2023). Therefore, MU activity analysis has become popular for applications such as brain–machine interfaces, recording paralytic motor activity (Gilja et al., 2015; Pandarinath et al., 2017), and other clinical/preclinical trials (Fraser et al., 2009; Todorova et al., 2014; Christie et al., 2015; Kao et al., 2017). Some research suggests that MU activity sometimes contains more accurate estimates of neural population dynamics (Sharma et al., 2015; Trautmann et al., 2019). However, it remains unclear which signals are retained, and which are lost, when one switches from SU to MU analysis.

The gaze control system provides an ideal model system to compare the SU versus MU codes, because spatiotemporal codes have already been described at the SU level. Neurons in higher-level gaze structures [superior colliculus (SC), lateral intraparietal cortex (LIP), FEFs, and supplementary eye fields (SEFs)] show separate visual and motor responses when a memory delay separates visual target presentation and saccade initiation (Gnadt et al., 1991; White et al., 1994; Umeno and Goldberg, 2001; Schall, 2015; Heusser et al., 2022). Further, these responses are spatially organized into visual and motor response fields (Schlag and Schlag-Rey, 1987; Andersen et al., 1992; Sommer and Wurtz, 2000, 2001; Schall, 2015; Hafed et al., 2023). Finally, although results vary with task, higher-level visuomotor structures primarily show eye-centered response fields (Andersen et al., 1985; Mullette-Gillman et al., 2005; Snyder, 2005; Park et al., 2006; Constantin et al., 2007; Keith et al., 2009; Caruso et al., 2018a,b). For example, the FEF and SEF, located in dorsolateral and medial frontal cortex, show predominantly eye-centered sensory/motor response fields for visual targets/saccades, respectively (Peck et al., 1995; Caruso et al., 2021).

**Figure 1.**
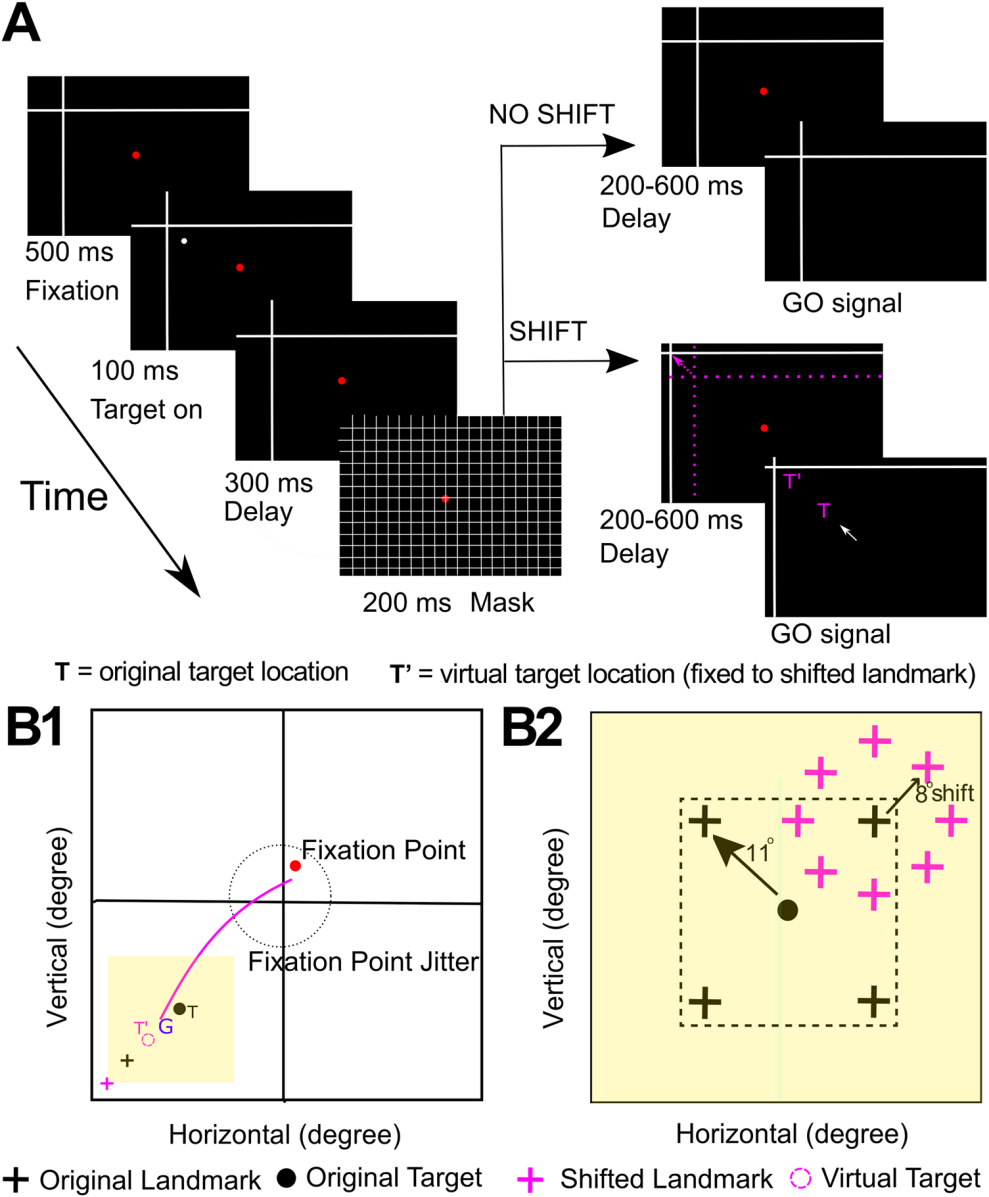
Representation of the experimental task and behavior. ***A***, Cue-conflict paradigm and its time course. The monkey began the task by fixating on a red dot for 500 ms while a landmark (white, intersecting lines) was present on the screen. Next, a target (white dot) was flashed for 100 ms, followed by a 300 ms memory delay where the target disappeared from the screen. After a 200 ms grid-like mask and another memory delay (200–600 ms), the monkey was cued by the disappearance of the fixation dot (i.e., go signal) to saccade head-unrestrained toward the remembered target location either when the landmark was shifted (represented by the broken pink arrow) or when it was not shifted. The monkey was rewarded when they placed their gaze (G) within a radius of 8–12° around the original target [at T (original target), T’ (virtually shifted target fixed to shifted landmark) or between T and T’]. The reward window was centered on T so that behavior was not biased. Note that the actual landmark shift was 8°, but it has been exaggerated in the schematic figure for clarity. The pink-colored objects are only used in the schematic figure for purposes of representation. ***B1***, Representation of a gaze shift (pink curve) from the initial fixation point (red dot) toward the virtual target (dotted pink circle) fixed to shifted landmark (pink cross). G represents the final gaze location, and the yellow square represents the area in ***B2***. The dotted circle stands for the jitter of the initial fixation points on the screen from one trial to another. ***B2***, Representation of all (four) possible landmark (black cross) locations and shifted landmark (pink cross, relative to the original landmark location) locations for an example target (black dot). The landmark was presented 11° around the original target in one of four oblique directions. The shifted landmark was presented 8° around the original landmark location in one of eight radial directions.

We have used a model-fitting approach (fitting various spatial models against response field data) to track sensorimotor transformations in the gaze system through time (Keith et al., 2009; Sadeh et al., 2015, 2018; Sajad et al., 2015, 2016). In contrast to decoding techniques, which allows one to extract information from noisy neural populations (Fraser et al., 2009; Meyers, 2013; Bremmer et al., 2016; Brandman et al., 2017; Dong et al., 2023), this technique tests the original spatial code used by individual neurons or populations (Keith et al., 2009; Burns et al., 2010; Drebitz et al., 2019; Ahmadi et al., 2021). Initial investigations in SC and FEF confirmed a distributed, eye-centered transformation from target (Te) coding in visual response fields to future gaze (Ge) coding in the saccade motor response. When a spatial “T–G” continuum between Te and Ge was used to track these codes through time, they showed a progressive transition from target to gaze coding at the population level (Sadeh et al., 2015, 2018; Sajad et al., 2015, 2016), even without a memory delay (Sadeh et al., 2020). But not all cells showed this transition: some visual response predicted Ge and some motor responses retained Te. Recently, this technique was applied to FEF/SEF response fields in the presence of background landmarks (Fig. 1*A*). Although background stimuli produced various modulations (Schütz et al., 2023), the data still showed a strong T–G transformation (Bharmauria et al., 2020, 2021). Therefore, we deemed this might provide an ideal dataset to test the relative robustness of sensorimotor codes derived from SU versus MU activity.

Here, we analyzed the spatial codes of SU and MU activity derived from the same FEF recording sessions (Bharmauria et al., 2020). After isolating SU and MU activity (Quiroga et al., 2004; Drebitz et al., 2019), we applied our model-fitting approach to test their visual and motor response fields. After confirming that SU/MU visual and motor population codes were best characterized overall by the Te and Ge models, respectively, we used the “T–G” spatial continuum between these models to characterize, quantify, and compare the spatiotemporal progression of SU versus MU activity coding schemes (Keith et al., 2009; DeSouza et al., 2011; Sadeh et al., 2015; Sajad et al., 2015). We also compared MU activity to “reconstructed MU activity” (the sum of SU activity derived from the same sites) to see if summed SU signals can trivially explain MU codes and sorted SU activity into regular and fast spiking neurons (Ding and Gold, 2012; Vinck et al., 2013; Bachatene et al., 2015; Bharmauria et al., 2015; Thiele et al., 2016; Dasilva et al., 2019; Trainito et al., 2019; Sajad et al., 2022) to determine if these cell types contribute differently to the visuomotor transformation.

We found that the MU and SU response fields show similar spatial codes and spatiotemporal transformations, but MUs show a “smoother” sensorimotor transition and lack certain nuances (i.e., gaze prediction in visual responses, residual target signals in motor responses, and different delay codes in the regular/fast spiking cells), resulting in a “cleaner” sensorimotor transition. We further show that these patterns are not replicated by summing SU activities, suggesting that meaningful information is lost in the process of spike sorting. We conclude that MU activity could provide a practical, quick, and reliable biomarker for basic sensorimotor transformations but may lack certain nuances important for the more cognitive aspects of sensorimotor behavior.

## Materials and Methods

Most experimental and analytic details of this study were published previously in analysis of SU response fields in the FEF (Bharmauria et al., 2020). These are summarized here along with specific details related to the MU activity response field analysis.

### Surgical procedures

All experimental protocols were approved by the Animal Care Committee of the University and were in alignment with the guidelines of Canadian Council on Animal Care on the use of laboratory animals. Neural data used in this study were gathered from two female *Macaca mulatta* monkeys (Monkey V and Monkey L). Two 3D search coils, each with a diameter of 5 mm, were implanted in the sclera of the left eye of the respective animal. The recording chambers in the FEF of both animals were implanted and centered at 25 mm anterior and 19 mm lateral. Surgeries were performed as previously done (Crawford et al., 1999; Klier et al., 2003). There was a craniotomy (19 mm diameter) beneath each chamber that allowed access to the right FEF. During the experiment, animals were placed in custom-designed chairs which allowed free head movements. To prevent animals from rotating in the chair, they wore a vest fixed to the chair. Furthermore, animals were placed in the setup with three orthogonal magnetic fields with two orthogonal coils mounted on their heads (Crawford et al., 1999). These fields induced a current in each coil. The amount of current induced by one of the fields is proportional to the coil's area parallel to this field. This allowed us to derive the orientation of each coil in relation to the magnetic fields and from this, eye orientations, head velocities, and eye and head accelerations (Crawford et al., 1999).

### Behavioral paradigm

We chose the following paradigm for the current study because it provides a visually rich, noisy dataset for extraction of spatial codes and has already been applied to an existing dataset of FEF recordings. We employed head-unrestrained gaze shifts (i.e., including coordinated eye saccades and head motion) to separate signals organized relative to eye, head, or space coordinates. Visual stimuli were presented using laser projections on a flat screen 80 cm away from the animal (Fig. 1*A*). The monkeys performed a memory-guided gaze task in which animals were trained to remember a target relative to a visual allocentric landmark (displayed using two intersecting lines). This led to a temporal delay between the target presentation and eye movement initiation. This allowed us to independently analyze visual (aligned to target) and eye movement-related (saccade onset) responses in the FEF. The experiments were conducted in a dark room to prevent any extraneous allocentric cues. Each trial began with the animal fixating on a red dot in the center of the screen for 500 ms in the presence of a landmark. A brief flash of the visual target (T, white dot) followed for 100 ms, then a 300 ms delay, followed by a grid-like mask for 200 ms (this concealed past visual traces and current and future landmarks), and a second memory delay (200–600 ms). The disappearance of the red fixation dot signaled the animal to perform a head-unrestrained saccade (indicated by the solid white arrow) toward the memorized location of the target either in the presence of a shifted landmark (broken pink arrow, 90% of trials shifted in one of the eight radial directions by 8°) or a nonshifted landmark (10%, zero-shift condition, i.e., landmark position did not change as before mask). The saccade targets were flashed one-by-one in a random manner throughout a neuron's response field. The details of the task are outlined in Figure 1*B1*,*B2*. In the shift condition, where the shifted target (T’) was fixed to the landmark, the animal was rewarded with a water-drop when its gaze (G) landed within 8–12° radius of the original target (animals were rewarded if they looked at T, T’, or anywhere in between). This large reward window is what allowed variability in the memory-guided gaze shifts and error accumulation (Gnadt et al., 1991; White et al., 1994; Sajad et al., 2015) and thus formed the logical base of our analysis (see below).

The response fields were tested roughly across horizontal and vertical dimensions (rectangular range of 30–80°). For both animals, there was a high correlation between the direction of the target and final gaze position in both dimensions [horizontal: Monkey V (*R* = 0.85), Monkey L (*R* = 0.83); vertical: Monkey V (*R* = 0.84), Monkey L (*R* = 0.89)]. This shows that the final gaze positions were aimed toward the vicinity of the target.

### Electrophysiological and behavioral recordings

**Figure 2.**
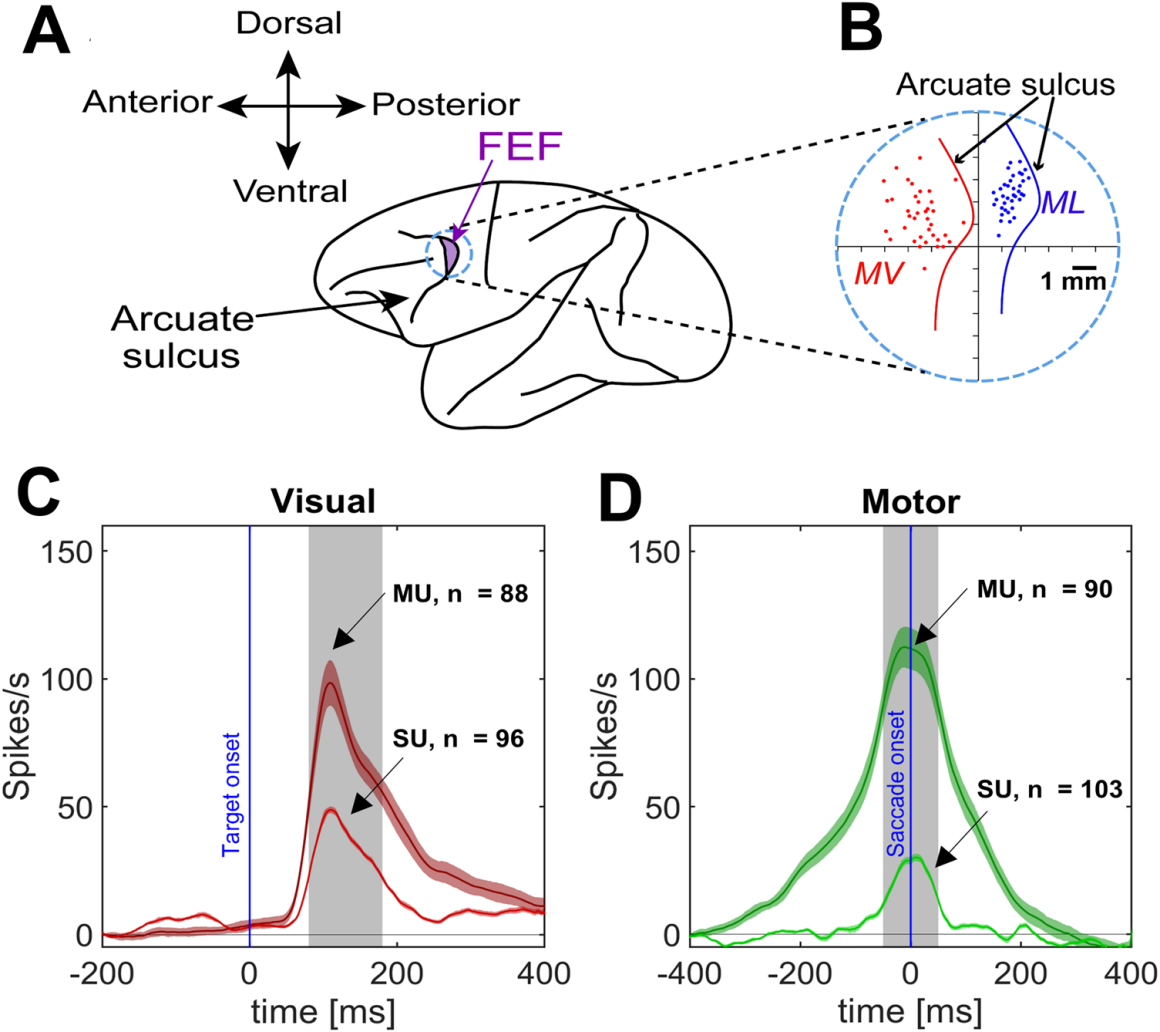
FEF recordings. ***A***, Acute FEF recordings were done with a single tungsten electrode. ***B***, Overlapped sites of neural recordings from two animals (Monkey V, MV and Monkey L, ML). Curved lines denote the arcuate sulcus. ***C***, Mean spike density profiles (with 95% confidence intervals) for SU (red) and MU (dark red) visual responses, obtained by averaging across the density plots for the top 10% activity at each recording site. Blue line indicates the target onset. ***D***, Same as ***C*** but for motor responses (SU light green, MU dark green). Blue line stands for saccade onset. Note: the top 10% activity from each neuron was taken and then pooled across all neurons. Note that the entire dataset (not just this top 10%) was used for our response field analysis.

We recorded acutely from the FEF, a gaze area associated with sensorimotor transformations (Siegel et al., 2015; Caruso et al., 2018b). Extracellular activity was recorded by lowering tungsten electrodes (0.2–2.0 mΩ impedance, FHC) into the FEF [using Narishige (MO-90) hydraulic micromanipulator] using the Plexon MAP System. The recorded activity was then digitized, amplified, filtered, and saved for off-line spike sorting (using template matching). The recorded sites (in head-restrained conditions) were further confirmed using a low-threshold electrical microstimulation (50 μA) as used previously (Bruce et al., 1985). A superimposed pictorial of the recorded sites from both animals is presented in Figure 2*A*,*B* (Monkey L in Blue and Monkey V in red). Figure 2, *C* and *D*, displays the overlapped and averaged spike density plots for top 10% (for each neuron and MU site) trials for the SU and MU, but the response field plots are plotted across all the trials for a single neuron. An average of 331 ± 156 (mean ± SD) trials/neuron were recorded. Mostly, the monkeys were free to scan the environment (head unrestrained) while neurons were searched for.

The experiment started once a neuron showed reliable spiking activity. Visual or motor response fields of neurons were characterized while the animals performed memory-guided gaze shifts. These gaze shifts started from an initial fixation location to a target (which was randomly flashed one-by-one, generally contralateral to the recording site) within an array (that varied between 4 × 4 to 7 × 7 depending on the size and shape of the response field) of targets (5–10° apart from each other). The initial fixation locations were jittered within a 7–12° window, which increased variability of 3D gaze, eye, and head distributions to initial fixation and the displacements. For our analysis, this variation allowed for separation between effectors and the space-fixed and eye-centered frames of reference for target and gaze (Keith et al., 2009; Sajad et al., 2020). There was no correlation between the initial gaze location and final gaze errors.

The 3D coil signals were sampled at 1,000 Hz to measure eye and head orientation in space (Bharmauria et al., 2020, 2021). Gaze (the 2D pointing direction of the eye in space) and eye-in-head positions were derived from these signals (Crawford et al., 1999). When conducting eye movement analysis, the saccade onset (eye movement in space) was marked as the point in time when the gaze velocity increased above 50°/s, while the gaze offset was marked as the point in time when the velocity decreased below 30°/s. The head movement was marked from the saccade onset up to the point in time when the head velocity decreased below 15°/s.

### Neural recordings and single-/multiunit activity sorting

The neural signal was first amplified by 50 and filtered with a bandwidth of 100–8 kHz. Then it was amplified again by 20 and filtered with another low-frequency bandwidth between 70 and 250 Hz. Finally, it was sampled at 40 kHz (A/D, analog-to-digital) to acquire the raw neural signal. We then manually isolated the SU activity from the saved neural recordings using template matching and further confirming it with principal component analysis (PCA) using the Plexon software. We only carried forward those neurons that had a relatively constant template throughout recording. MU activity refers to the entirety of neural signals detected by an electrode, corresponding to activity from multiple neurons within the surrounding tissue. Since it is not possible to position the electrode near all of those neurons at the same time, MU activity signals tend to have a lower amplitude compared with SU signals (i.e., the difference between the recorded voltage signals) where “big” signals close to the electrode are typically selected.

We rethresholded the high-pass filtered raw neural signal to isolate the MU activity with a spike detection threshold of 3.5 standard deviations (SDs) above the mean noise level, meaning that any neural spike surpassing this threshold is considered a significant spike, indicative of genuine neural activity rather than random noise. According to previous reports (Buzsáki, 2004; Quiroga, 2012; Rey et al., 2015), the MU activity was composed of neurons that were most likely within the vicinity of ∼100–200 μm around the electrode tip, compared with ∼50 μm for the SU signals. This procedure allowed us to discriminate the spikes above the noise level (Bullock, 1997; Buzsáki, 2004; Burns et al., 2010; Mattia et al., 2010; Land et al., 2013).

To reconstruct MU activity from SU activity (for comparison with the original MU data), we only summed activity from SUs extracted from the same site and recording session. This was necessary to ensure that the behavioral conditions were identical for model fitting, for an “apples-to-apples” comparison with the corresponding original MU data.

### Data inclusion criteria and sampling window

Only neurons (and the corresponding MU sites) that were clearly isolated and task-modulated were analyzed, i.e., SU/MU with clear visual activity and/or with perisaccadic movement (Fig. 2*C*,*D*). Neurons with only postsaccadic activity (activity after the saccade onset) or that lacked significant spatial tuning (see “testing for spatial tuning” below) were excluded from the analysis. While trials where monkeys landed their gaze within the reward acceptance window were included, we excluded trials when the gaze end points went beyond 2° distance from the average gaze endpoint for a given target. In conducting neural activity analysis, the “visual epoch” referred to a fixed 100 ms window of 80–180 ms aligned to the target onset and the “movement period” referred to a high-frequency perisaccadic 100 ms (−50 to +50 ms relative to saccade onset) window (Sajad et al., 2015). This allowed us to have a good signal-to-noise ratio for neuronal activity analysis and most likely corresponded to the epoch during which gaze shifts influenced neural activity.

### Fitting neural response fields against spatial models

In order to test between different spatial models, they must be experimentally separable (Keith et al., 2009; Sajad et al., 2015). For instance, in our paradigm, the variability induced by memory-guided gaze shift end points allows one to discriminate target coding from the gaze coding, natural variations in initial eye and head locations allow one to distinguish between different egocentric reference frames, and variable eye and head components for the same gaze shift permit the separation of different effectors (Crawford et al., 1999; Keith et al., 2009). In our method, we use these experimentally derived measures as coordinate systems to analyze neural response field data and then discriminate which spatial model best describes the data.

**Figure 3.**
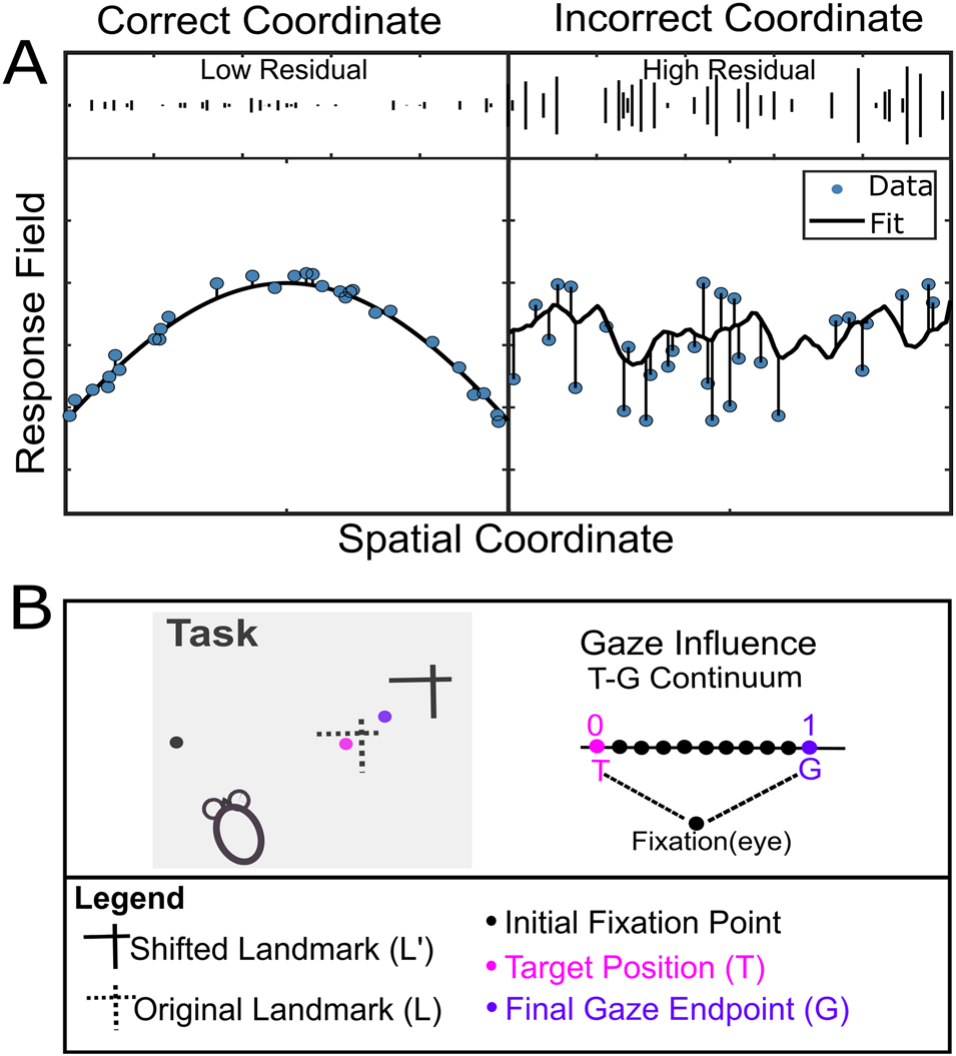
Schematic representation of spatial model-fitting procedure. ***A***, Illustration of the spatial model-fitting technique. The *x*-axis indicates target or gaze position along one axis (i.e., horizontal or vertical) of the spatial coordinate system, and the *y*-axis is the associated neural activity. If the activity (blue data points) related to a fixed position is plotted in the correct coordinate frame (left), it yields lower residuals. On the other hand, activity that is plotted in an incorrect coordinate frame leads to higher residuals (right). ***B***, Left, Illustration of the task and gaze influence for an example trial. The black dot depicts the initial gaze fixation point, and the pink dot represents the target position (T) in relation to the original landmark (L) position (dotted intersecting lines). The solid intersecting lines. The purple dot depicts the final gaze endpoint (G), shifted partway with the shifted landmark (L’, solid lines). Right, An egocentric T–G continuum was used to test the optimal point coded by each MU/SU between T and G along ten uniform steps. See Extended Data Figure 3-1 for more details.

The logic of our response field fitting in different reference frames is schematized in Figure 3*A*. If the response field data is plotted in the correct/best coordinate system, this should yield a uniform distribution of data with low residuals (i.e., errors between the actual data and a mathematical fit made to these data; Fig. 3*A*, left). On the other hand, if the coordinate system/fit does not match the data well, this will lead to higher residuals (Fig. 3*A*, right). For example, if an eye-fixed response field is computed in eye coordinates, this should yield lower residuals compared with head or space coordinates (Sajad et al., 2015). For the actual analysis, we employed a nonparametric fitting method to characterize the neural activity with reference to a specific spatial coordinate system and varied the spatial bandwidth (kernel 2–25°) of the fit to plot any response field size, shape, or contour (Keith et al., 2009). We tested various spatial models using predicted residual error sum of squares (PRESS) statistics. In this method, the residual for a trial is computed by comparing the actual activity relative to fits computed from other trials (similar to cross-validation). For a given neuron and temporal window (Fig. 2), this was done for all trials, for each bandwidth of fit and for each spatial model tested (e.g., Te, Ge, etc.). The model and bandwidth that yield the lowest residuals were deemed “best.” The PRESS residual of this model was then compared with the PRESS residuals of the other models at the same kernel bandwidth using a two-tailed Brown–Forsythe test to test for significant differences in the goodness of fit (typically, a significantly superior fit is only found in a fraction of individual neurons). Finally, we performed the same statistical analysis (Brown–Forsythe) at the population level by comparing the means of PRESS residuals across neurons (Keith et al., 2009; DeSouza et al., 2011; significance is more often reached at this level).

10.1523/ENEURO.0413-23.2024.f3-1 Figure 3-1 **A**, Basic egocentric reference frames: eye (e), head (h) and body / space (s). **B**, Different variables, and egocentric models for an example trial. Models tested: the difference between the initial and the final head orientation relative to space (dH); the difference between the initial and the final eye orientation relative to the head (dE); future orientation of the head in space coordinates (Hs); Eye in head (Eh); Future gaze in space (Gs); Future gaze in head (Gh); Future gaze in eye (Ge); Target in space (Ts); Target in head (Th); Target in eye (Te). Download Figure 3-1, TIF file.

### “Cardinal” models tested

We first repeated the model-fitting analysis of the previous study (Bharmauria et al., 2020) on SU and MU activity populations to confirm if Te and Ge were the overall best “canonical” models to represent response field activity in this task. [Note that in the recent investigation (Schütz et al., 2023), these models were called *T_F(e)_*/*G_F(e)_* to specify the foveal coordinate origin (0,0), but this can be assumed here.] Again, these are not theoretical models but rather models that were derived from the experimental measures described above. Possible egocentric models included target position versus effector (gaze, eye, head) displacement or position in three egocentric frames (see Extended Data Fig. 3-1 for schematic representations). Specifically, target in space, eye, or head coordinates (Ts, Te, Th); future gaze in eye, head, and space coordinates (Ge, Gh, Gs); eye displacement (dE), i.e., the difference between initial and final eye orientation relative to the head; head displacement (dH), i.e., the difference between initial and final head orientation relative to space; and finally, future orientation of the head in space coordinates (Hs; we removed dG because it was indistinguishable from Ge in this dataset). We did not test allocentric models (e.g., target relative to landmark, landmark relative to eye) here because they were statistically eliminated at the population level in previous studies (Bharmauria et al., 2020, 2021; Schütz et al., 2023), and our goal here was to test the population egocentric visuomotor transformation.

### Intermediate spatial models: the TG continuum

Target position and final gaze position were rarely in exactly the same place, in part because of systematic and variable endogenous errors and in part (in this dataset) because the landmark shift caused G to shift partway in the same direction (Fig. 3*B*, left). Previous studies suggested that FEF responses do not exactly fit against spatial models like Te or Ge but actually may fit best against intermediate models between the canonical ones (DeSouza et al., 2011; Sadeh et al., 2015; Sajad et al., 2015, 2016). The spatial continuum between Te and Ge (“T–G continuum”) provided the best overall description of the data and a signature for the sensorimotor transformation, where G includes variable errors relative to T (Sajad et al., 2015). This continuum was created by taking 10 (10%) steps between and beyond Te and Ge (Fig. 3*B*, right). Here, these T–G fits were then done at each of these steps, and where the fit yields the lowest PRESS residuals was considered “best”, as described above.

Note that even the best model would not be expected to have zero residuals, because of uncontrolled factors such as biological/measurement noise, nonspatial factors such as arousal/motivation levels (Sajad et al., 2020), and in the case of the motor response, dynamics of the movement (van Opstal, 2023).

### Testing for spatial tuning

The method described above assumes that neuronal activity is structured as spatially tuned response fields. Other neurons might implicitly subserve the overall population code (Bharmauria et al., 2014, 2016a; Goris et al., 2014; Leavitt et al., 2017; Chaplin et al., 2018; Zylberberg, 2018; Pruszynski and Zylberberg, 2019), but here, we were testing for explicit codes. Therefore, we confirmed the spatial tuning of neurons before including them in further analysis. We tested spatial tuning by randomly shuffling (100 times to obtain random 100 response fields) the firing rate data points across the position data that we obtained from the best model. The mean PRESS residual distribution (PRESS_random_) of the 100 randomly generated response fields was then statistically compared with the mean PRESS residual (PRESS_best-fit_) distribution of the best-fit model (unshuffled, original data). If the best-fit mean PRESS fell outside of the 95% confidence interval of the distribution of the shuffled mean PRESS, then the neuron's activity was deemed spatially selective. At the population level, the percentage of neurons showing significant spatial tuning varied over time. Thus, we removed the best fit values where the populational mean spatial coherence (goodness of fit) was statistically indiscriminable from the baseline (before target onset). We then defined an index (coherence index, CI) for spatial tuning. The CI for an SU/MU site was calculated according to a previous report (Sajad et al., 2015), as shown below:

$$\mathrm{Coherence}\;\mathrm{index}=1-\left(\frac{\mathrm{PRES}S_{\mathrm{bestfit}}}{\mathrm{PRE}S_{\mathrm{random}}}\right)\;;$$

if the PRESS_best-fit_ was similar to PRESS_random_, then the CI would be roughly 0, whereas if the best-fit model is a perfect fit (i.e., PRESS_best-fit _= 0), then the CI would be 1. We only included those SU/MU sites in our analysis that showed significant spatial tuning.

### Time normalization for spatiotemporal analysis

In this task, the time between the onset of the target and the gaze onset was not equal across trials because of a variable delay period. To accommodate this variable trial time for transformations between these events, we time-normalized the duration of each trial (Sajad et al., 2016; Bharmauria et al., 2021). This procedure was conducted only for the spatiotemporal analysis presented near the end of the results section (Fig. 11 and Extended Data Figs. 10-1, 11-1). Accordingly, the neural activity across different trials (between the events that we were interested in—80 ms after the target onset until the onset of saccade for the whole population in Fig. 11) was divided into 14 equal half overlapping bins (with a range of 112.26–178.93 ms depending on the trial). If we aligned the trials in a standard way (relative to visual stimulus onset or saccade onset), this would lead to the loss and/or mixing of neural activities across trials. The rationale behind the bin number choice was to make sure that the sampling time window was wide enough, and therefore robust enough, to account for the stochastic nature of spiking activity. During this normalization procedure, the duration of the mask was also convolved in time (on average from step 4.69 to step 7.63). Therefore, the time-normalization procedure was performed to account for the different delay period and the details of this procedure are provided in the previous study (Sajad et al., 2016). For this analysis, the neural firing rate (in spikes/second; the number of spikes divided by the sampling interval for each trial) was sampled at 14 half overlapping time windows from these time-normalized data. The choice for sampling window numbers was based on the approximate ratio of the duration of the visual burst to delay period to movement burst (Sajad et al., 2016). The final (14th) time step in Figure 11 also contained some part of the perisaccadic sampling window as mentioned above. This time-normalization procedure allowed us to consider the entire time-period (across different trials) of visual–memory–motor responses as an aligned spatiotemporal continuum.

### Code availability statement

All codes are custom-written and available on request.

## Results

In the current study, we compared sensorimotor codes by fitting spatial models against SU and MU activity derived from anatomically matched FEF populations. In summary, we recorded task-related neural activity from 257 FEF sites in both animals and isolated 312 FEF units. After applying our exclusion criteria, including tests for spatial tuning, 147 SUs (including visual, V; visuomotor, VM; and motor, M neurons) and the corresponding 103 MU sites, respectively, were taken forward for analysis. Of these, 91 SU sites were accompanied by MU data and 59 MU sites were accompanied by SU data for motor responses (the difference is due to the exclusion criteria). Sixty-three SU sites were accompanied by MU data, and 52 MU sites were accompanied by SU data for visual responses. The anatomic distributions of the SU and MU sites were nearly identical in both animals (Fig. 2*B*). Finally, of the 147 SU sites, we analyzed 102 visual and 109 motor responses; and of the 101 MU sites, we analyzed 88 visual and 90 motor responses.

### Summary of overall statistical analysis of egocentric models

As a preliminary step, we first tested the Te and Ge models (the overall preferred models for sensory and motor activity in our previous study) against other potential egocentric models (target, eye, head, and gaze displacement or position coding in various egocentric frames of reference). Again, these tests are based on finding the model that yields the overall lowest residuals between our nonparametric fits and actual response field data and the testing for significance relative to other models.

**Figure 4.**
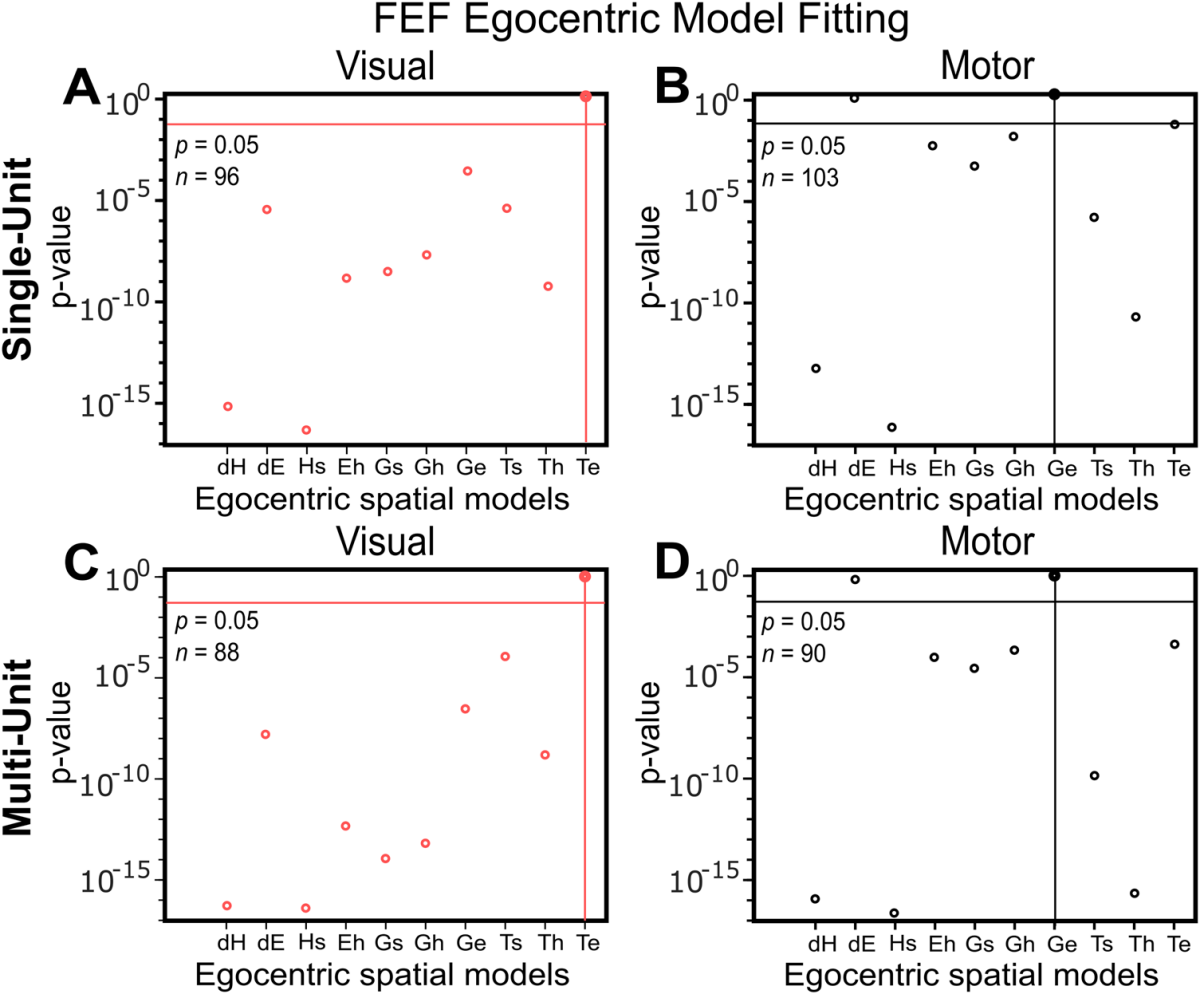
*p* value statistics performed on the residuals of different egocentric models (Brown–Forsythe test) in FEF SU ***A***, visual and ***B***, motor responses. Same analysis for MU ***C***, visual and ***D***, motor responses. Te is the best model for the visual response and Ge is the best model for the motor response in both the SU and MU activities. Best model: *p* = 10^0 ^= 1; the best-fit spatial model that yielded the lowest residuals. The horizontal line indicates *p* = 0.05. Models tested: the difference between the initial and the final head orientation relative to space (dH); the difference between the initial and the final eye orientation relative to the head (dE); future orientation of the head in space coordinates (Hs); eye in head (Eh); future gaze in space (Gs); future gaze in head (Gh); future gaze in eye (Ge); target in space (Ts); target in head (Th); target in eye (Te).

**Figure 5.**
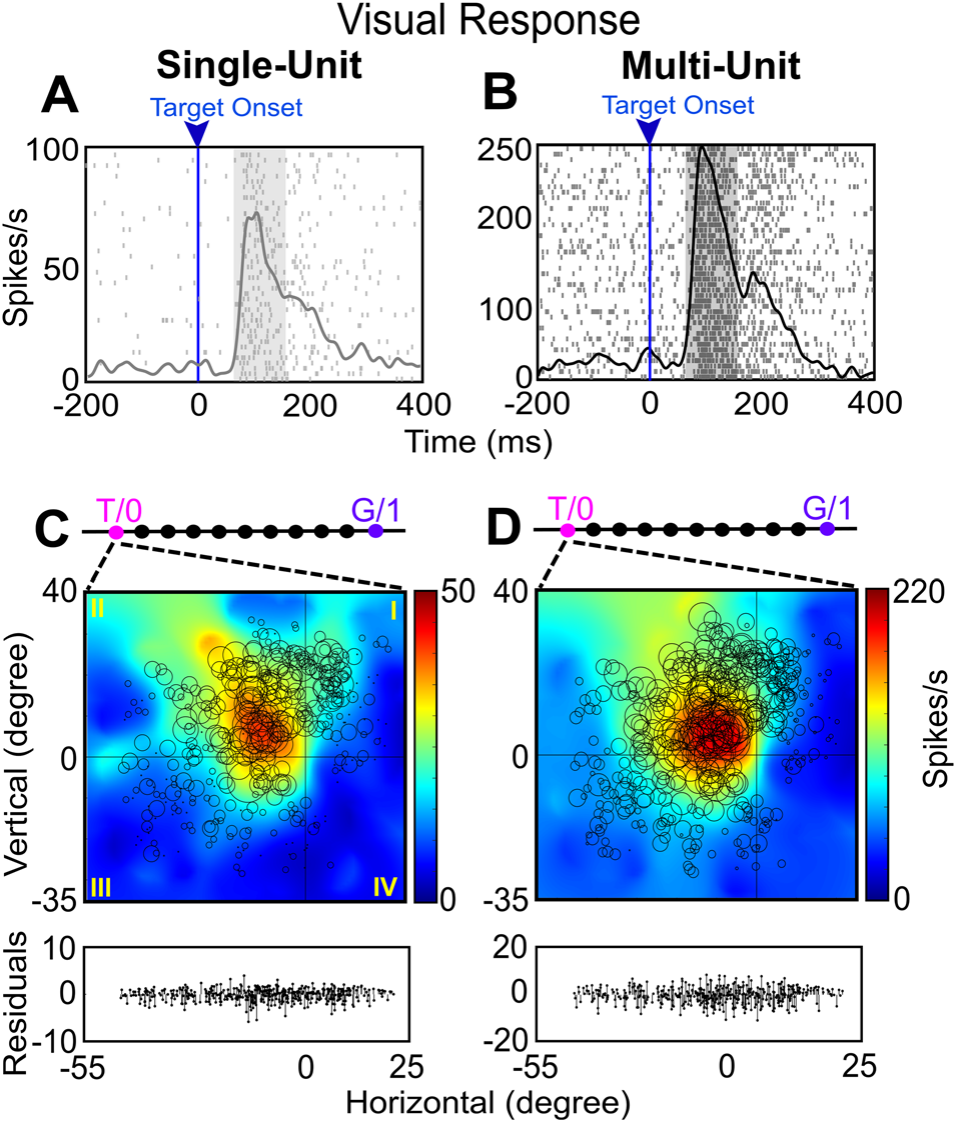
Examples of SU and MU visual response field analysis. ***A***, ***B***, Raster/spike density plot (with top 10% of responses) of an SU and MU visual response aligned to target onset (blue arrow), respectively. The shaded gray region represents the sampling window (80–180 ms) for the response field analysis. ***C***, ***D***, Representation of the response field of SU and MU visual activity along the T–G continuum, respectively. The size of the circle corresponds to the magnitude of the response and the heat map represents the nonparametric fit to the data (red area is the response field hotspot). The converging dotted lines indicate that both SU and MU fit best at exactly at T. 0,0 indicates the center of the coordinate system which yielded the lowest residuals (best fit). The graph at the bottom of ***C*** and ***D*** represents the respective residuals for the response field plots. The roman numerals in ***C*** indicate the quadrants.

**Figure 6.**
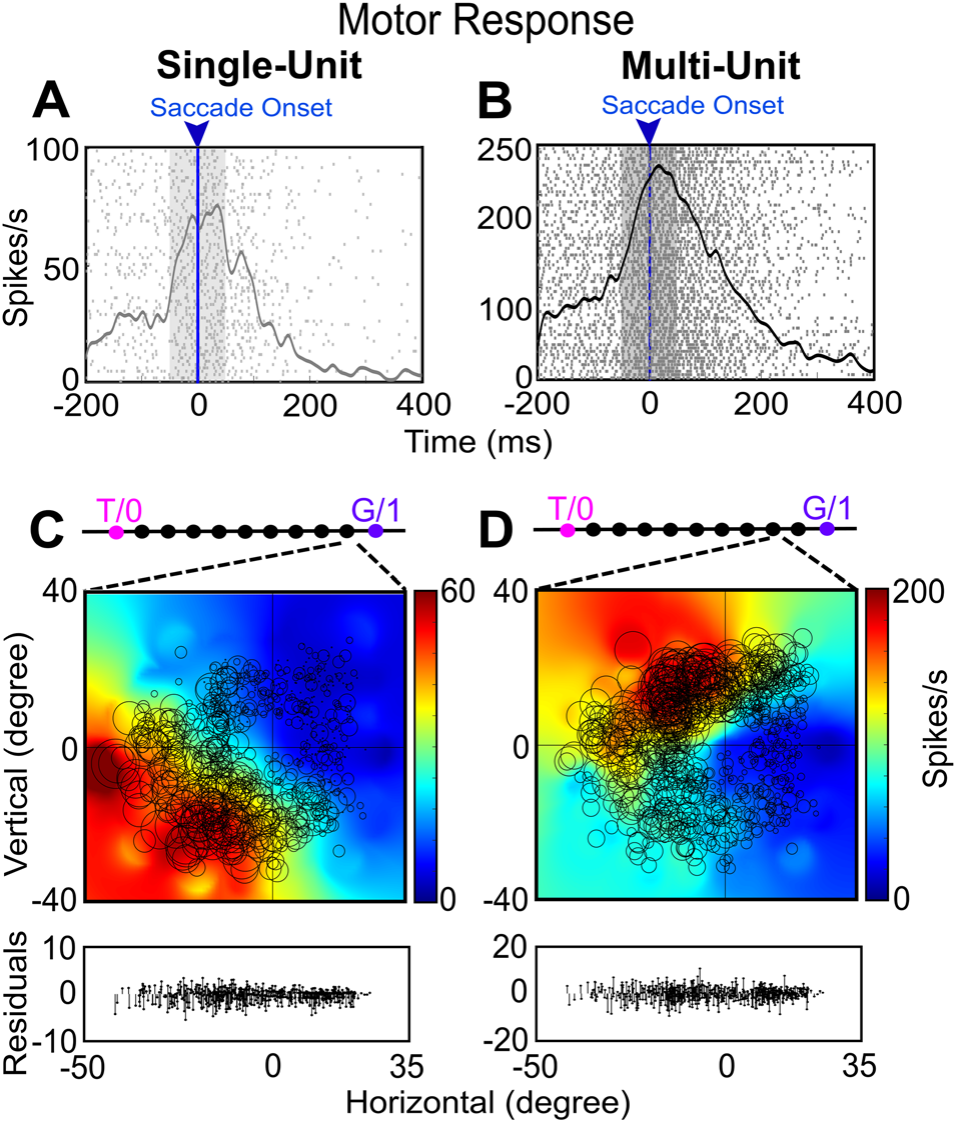
Examples of SU and MU motor response field analysis. ***A***, ***B***, Raster/spike density plot (with top 10% of responses) of an SU and the corresponding MU motor response aligned to the saccade onset (blue arrow), respectively. The shaded gray region is the sampling window (−50 to +50 ms) for the response field analysis. ***C***, ***D***, Representation of the response field of SU and MU for motor response along the T–G continuum, respectively. The SU response field fits best at the ninth step from T and MU response field fits best at the eighth step. The graph at the bottom of ***C*** and ***D*** represents the respective residuals for the response field plots.

Figure 4 summarizes this result for both our SU (Fig. 4*A*,*B*) and MU (Fig. 4*C*,*D*) populations (see Figs. 5, 6 below for example response field fits). As expected, residuals for the MU fits were significantly larger (Mann–Whitney *U* test, *p* < 0.001) than the SU residuals (for both visual and motor responses), because they required fits to multiple response fields with different baselines and peaks. However, the overall patterns of best model fits for the SU and MU populations were similar: the visual response best codes for Te and the motor response best codes for Ge, as found previously in the SU activity study (Bharmauria et al., 2020). Data points below the horizontal line (*p* = 0.05) indicate models with worse fits. In the case of the visual responses, all other models were significantly eliminated in both population datasets. In the case of the motor responses, all models were significantly eliminated except for gaze displacement (dG, which is very similar to Ge in this dataset; see Materials and Methods) and eye-in-head displacement [dE; also very similar to Ge when head contribution to gaze is small (Klier et al., 2001)]. This does not mean that these populations do not code for other variables in individual cells or in more subtle ways (Schütz et al., 2023) but shows that Te and Ge provide the best overall measures of their explicit population coding scheme. These results confirm the previous SU analysis and tend to suggest that MU populations show similar results. To further investigate this, a more in-depth analysis was performed based on the T–G continuum between the two best models.

### Example comparisons of single-unit and multiunit activity response fields along the T–G continuum

Previously, it was shown that the major egocentric transformation occurs between target-to-gaze (T–G) by plotting response fields at intermediate steps between a T–G continuum (Bharmauria et al., 2020). Here, a similar analysis on the MU activity was done to compare it with the SU activity. Figure 5 shows a typical visual response field analysis along the T–G continuum for SU and MU activity recorded at the same site. Figure 5, *A* and *B*, shows the raster and spike density (gray and black curves; representing top 10% of responses) of an SU and the corresponding MU visual response aligned to the target onset (blue line and arrow), respectively. The shaded gray region is the sampling window (80–180 ms) for the response field analysis. The SU response (firing frequency along the *y*-axis) is considerably lower than the MU response (because this combined several neurons).

Figure 5*C* shows the best SU fit for the visual response field along the T–G continuum. The response field best fits at T as indicated by the converging broken lines. Each circle represents the response to one trial, where the bigger the circle, the higher the response is. The heat map represents the nonparametric fit to the data. Note that the main cluster of large circles (and the corresponding red “hotspot” of the fit) is located in the upper-left quadrant relative to the center (0,0, i.e., initial fixation). This localized peak denotes the typical “closed” response field often seen in FEF visual data. The residuals (difference between the fit and each data point) are shown below the response fields.

Figure 5*D* shows the response field map of the corresponding MU, again with the best fit at T. In this case, the response fields of SU and MU activity were very similar (both showing similar “closed” organization at the same location), with the same best fits (T), and their fit residuals were qualitatively similar. Overall, this suggests that SU and MU activity might carry similar spatial information, but we will document this more thoroughly below.

Figure 6 shows a typical example of an SU and MU motor response field analysis from the same recording site. Note: two SUs were extracted from this site, whereas only one of these is shown in Figure 6*A*,*C*. Figure 6, *A* and *B*, shows the raster and spike density (same conventions as Fig. 5 for visual analysis) of an SU (Fig. 6*A*) and MU activity (Fig. 6*B*) for a motor response aligned to the saccade onset (blue line and arrow).

Figure 6*C* shows an example motor response field fit of an SU along the T–G continuum. In this case, both the SU and MU response fields showed the typical “open” pattern, with activity continuing to increase away from the center of the coordinates but peaking in different quadrants (lower-left for SU activity, upper-left for MU activity). This suggests that the response field of MU was strongly influenced by nearby neurons (the second neuron, not shown, isolated from the same site had a response field hotspot in the second quadrant). However, both the SU and MU activities showed a similar best fit point along the T–G continuum: one step from G (for SU activity) and two steps from G (for MU activity), i.e., close to Ge. In short, despite their differences, both the SU and MU motor responses utilized a similar code (future gaze relative to initial gaze). Collectively, the above examples suggest that MU and SU activities carry comparable information.

### Population analysis along the T–G continuum

**Figure 7.**
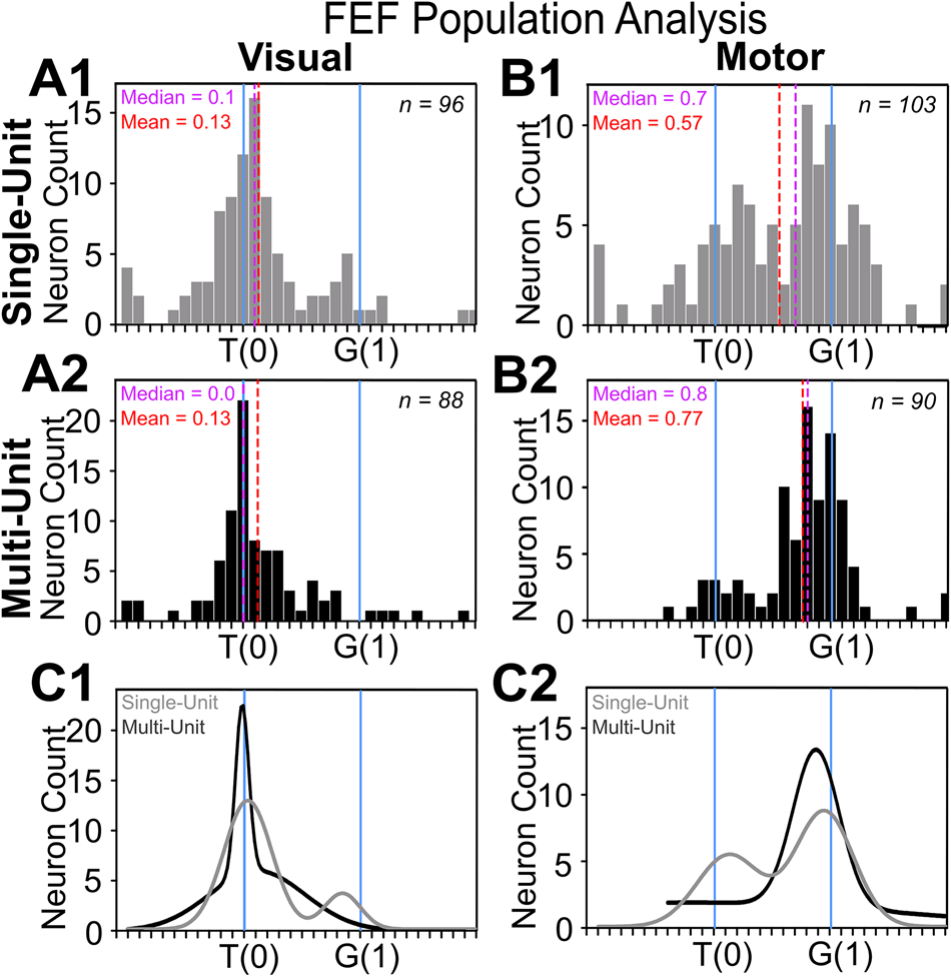
Frequency distribution of SU and MU along the T–G continuum at the population level. ***A1***, Frequency distribution of all spatially tuned SU visual responses (*n* = 96) with the best fit closer to T (mean = 0.13; median = 0.1). ***A2***, Frequency distribution of all spatially tuned MU visual responses (*n* = 88) with the best fit closer to T (mean = 0.13; median = 0). ***B1***, Frequency distribution of all spatially tuned SU motor responses (*n* = 103) with a shifted distribution toward G (mean = 0.57; median = 0.7). ***B2***, Frequency distribution of all spatially tuned MU motor responses (*n* = 90) with a significantly shifted distribution toward G (mean = 0.77; median = 0.8). ***C1***, The sum of two Gaussian distributions of SU (two tuning curves) and MU (one tuning curve) visual responses. ***C2***, The sum of two Gaussian distributions of SU (two tuning curves) and MU (one tuning curve) motor responses. In both cases, the smaller secondary peak disappeared.

To quantify how representative the above examples were, we plotted the overall distribution of best fits for the SU and MU populations. Figure 7*A1* shows the distribution of best fits for the SU visual responses (*n* = 96) along the T–G continuum with a primary peak around T and a secondary smaller peak near G. Most units (81.25%) showed a best fit below 0.5 (i.e., closer to T than G) and conversely, 18.75% showed a best fit closer to G. Overall, the population distribution showed a best fit around T but was significantly shifted toward G (mean = 0.13; median = 0.10; *p* = 0.03; one-sample Wilcoxon signed rank test, tested against a value of 0 for T). This suggests that the SU visual responses primarily encoded the target, but some units already predicted the future gaze location.

In comparison, the MU visual distribution (*n* = 88; Fig. 7*A2*) retained the primary peak near T. Although the overall shift from T toward G was still significant (mean = 0.13; median = 0; one-sample Wilcoxon signed rank test *p* = 0.013), the secondary “G” peak in SU disappeared in the MU fits. Likewise, the number of best fits closer to G than T decreased to 15.9%. Overall, these results suggest that the MU visual response, comprising several closely connected neurons, better reflected target position whereas gaze prediction was attenuated.

Figure 7, *B1* and *B*2, shows similar distributions for motor responses. Overall, the SU motor distribution was significantly shifted toward G compared with the SU visual distribution (*p* < 0.0001; Mann–Whitney *U* test). As expected, the SU motor (*n* = 103) distribution had a primary peak near G but still had a considerable secondary peak near T. In total, 44.6% of SU motor responses showed best fit scores below 0.5 (closer to T than G), suggesting considerable retention of target information in the motor response. As a result, the overall SU distribution was significantly shifted from both T and G toward an intermediate point (mean = 0.57; median = 0.7; *p* < 0.0001; one-sample Wilcoxon signed rank test).

The MU motor distributions (*n* = 88; Fig. 7*B2*) also showed a significant shift toward G (*p* < 0.0001; one-sample Wilcoxon signed rank test) and a strong “G” peak, but the secondary peak was attenuated: now only 17.77% of the fits were closer to T than G. As a result, the overall shift in MU (mean = 0.77; median = 0.8) was larger than the SU shift (mean = 0.57; median = 0.7). In other words, MU activity possessed a better canonical gaze code than SU activity but retained less information about the original target location.

Figure 7, *C1* and *C2*, superimposes the SU and MU Gaussian distributions for the visual and motor fit frequency distributions shown above. Overall, there was no significant difference between the entire SU and MU distributions for either visual (*p* = 0.922; Mann–Whitney *U* test) or motor responses (*p* = 0.063; Mann–Whitney *U* test). However, the variances of the fit distributions were significantly different for SU versus MU motor comparison (*F* = 2.19; *p* = 0.0002). Further, in both cases (visual and motor responses), the primary peak (T for visual, G for motor) is similar for SU and MU, but the secondary peak (G for visual, T for motor) is attenuated or missing in the MU population. Consistent with this, the proportion of SU fits below 0.5 (closer to T) in the motor response was significantly less than that of the equivalent MU fits (*z*-test for proportions; *z* = 3.63; *p* = 0.0028). Collectively, these data support the notion that MU visual and motor activities encode purer representations of T and G, respectively, whereas the secondary SU codes (predictive G in the visual response and retained T in the motor response) were attenuated.

### Visuomotor transformations within SU/MU response fields

**Figure 8.**
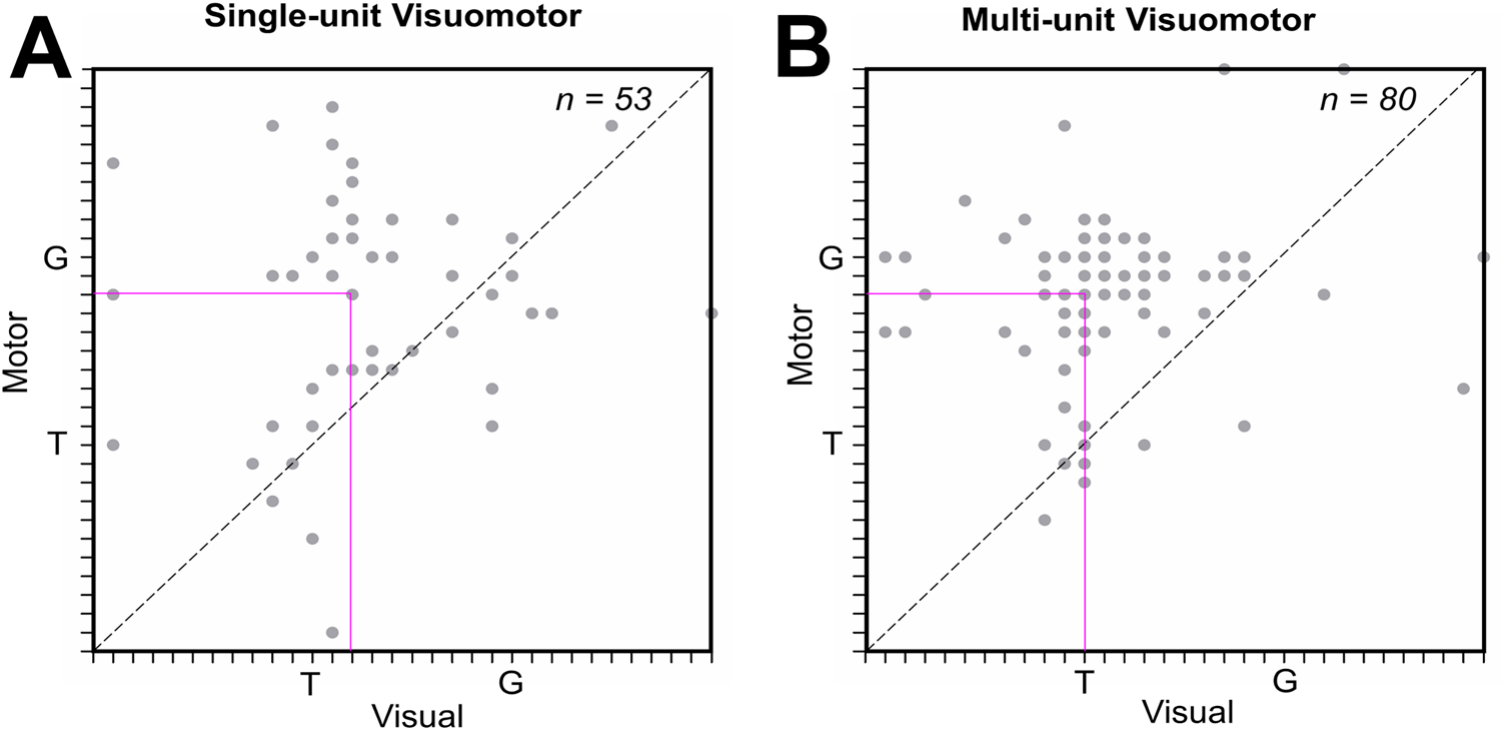
Visuomotor transformations within single- and multiunit response fields along the T–G continuum. Movement best fit (*y*-axis) as a function of the corresponding visual best fit (*x*-axis) for VM neurons (***A***) and corresponding MU activity (***B***) sites. Each dot represents one neuron/site. The pink intersecting lines indicate the median of the best fit for the movement against the median of the best fit for visual responses. The distribution was significantly shifted toward G in both cases (*p* < 0.0001, Wilcoxon matched-pairs signed rank test) suggesting visual-to-motor transformation from the target (T) to gaze (G) coding in both cases. However, their (***A*** and ***B***) slopes and elevations were not significantly different from each other, implying a similar profile of visual-to-motor transformations. See Extended Data Figure 8-1 for more details.

It is possible that the overall population shift from target to gaze coding could occur only due to transference of activity from visual to motor cells, as it also occurs within cells in SU activity (Sadeh et al., 2015; Sajad et al., 2015). To test if MU activity shows the same behavior, we contrasted visual and motor codes for neurons/sites that have both visual and motor responses (Fig. 8). Figure 8*A* shows the motor best fits (*y*-axis) as a function of the visual fits (*x*-axis) for individual neurons. Figure 8 illustrates the motor best fits (*y*-axis) as a function of the visual fits (*x*-axis) for the MU sites. An upward/leftward shift in these data suggests a transformation from target to gaze coding within visuomotor cells (Sadeh et al., 2015; Sajad et al., 2015). In both cases (SU and MU activity), most of the points and the intersection of medians (pink lines) were above the line of unity, and this was significant at the population level (*p* < 0.0001; Wilcoxon matched-pairs signed rank test) suggesting a visual-to-motor transformation in both cases. Further, the slopes (*F* = 0.23; *p* = 0.88) and elevations (*F* = 0.70; *p* = 0.40) for SU and MU distributions were not significantly different from each other, suggesting that SU and MU activities reveal similar within-cell/site visuomotor transformation.

10.1523/ENEURO.0413-23.2024.f8-1 Figure 8-1 **Site-matched analysis. A,** Visual MU sites as a function of the corresponding best fit score of visual SUs. No significant correlation was observed (Spearman R = 0.05, Slope = - 0.04 ± 0.08, p = 0.66). **B,** Motor MU sites as a function of the corresponding best fit score of motor SUs. A modest correlation (Spearman R = 0.20, Slope = 0.10 ± 0.06) but nearly reaching significance was observed (p = 0.053). Download Figure 8-1, TIF file.

### Site-matched anatomic comparison: multiunit versus single-unit

We next tested if these fit distributions were comparable for anatomically matched recording sites. Note that the anatomic variance could arise from both 2D distribution of recordings as well as cortical layer depth, which we could not measure accurately. For this analysis only specific sites that contained both SU and MU fits were obtained for visual and/or motor responses, taking the values of the SU fits against the MU fits. Based on Figure 7, we hypothesized that SU and MU spatial codes might show some anatomical relation, since there was no significant difference in their overall distributions. However, we did not find significant correlation (Extended Data Fig. 8-1) for MU versus SU for visual responses for anatomically matched sites (Spearman *R* = 0.05; slope = −0.04 ± 0.08; *p* = 0.66). A modest (Spearman *R* = 0.20; slope = 0.10 ± 0.06) but insignificant (*p* = 0.053) correlation was observed for motor responses (Extended Data Fig. 8-1). Overall, this suggests that variations in the SU and MU fits do not reflect anatomy.

### Multiunit activity versus reconstructed summed activity

**Figure 9.**
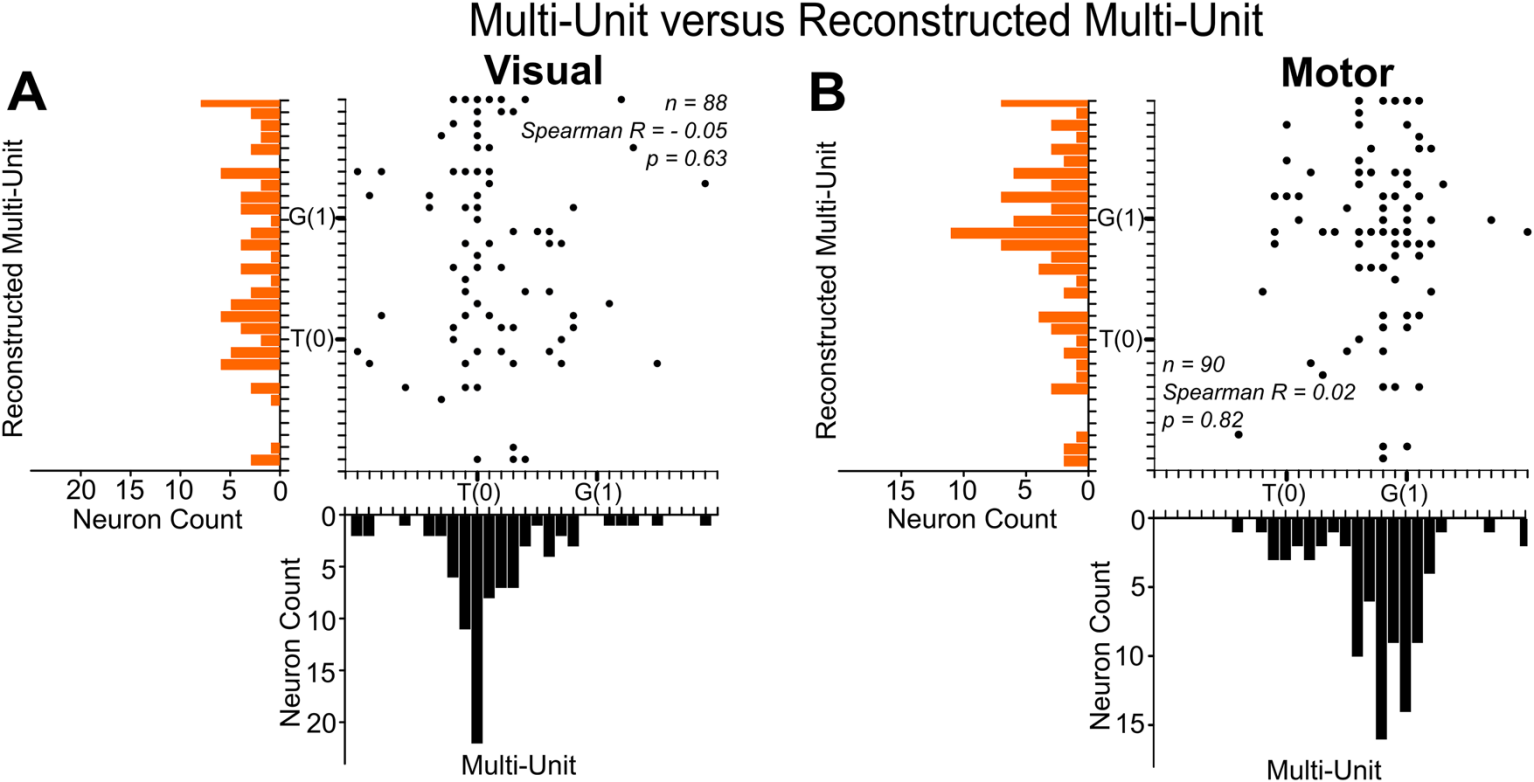
Reconstructed MU versus original MU comparison. ***A***, Reconstructed MU best fit scores as a function of site-matched original MU best fit scores for visual responses (*n* = 88). No correlation (Spearman *R* = −0.05; slope = −0.23 ± 0.18; *p* = 0.63) was observed between the reconstructed MU and original MU sites. ***B***, Reconstructed MU best fit scores as a function of site-matched original MU best fit scores for motor responses (*n* = 90), with no significant correlation (Spearman *R* = 0.02; slope = 0.11 ± 0.20; *p* = 0.82).

The retention of the primary visuomotor code in MU activity described above poses the question: is this pattern simply caused by summed activity of several SUs, or do other signals removed in spike sorting play a role? To test this, we reconstructed a population of MUs by combining all of the neurons (whether tuned, untuned, unresponsive, or postsaccadic) from each original MU site (*n* = 103) and repeated our analysis along the T–G continuum for the visual (*n* = 88) and motor (*n* = 90) MU sites (Fig. 9*A*,*B*). A total of 204 neurons were included for this analysis across 103 sites. If the MU signal is simply the sum of SU signals, one would expect the reconstructed dataset (without additional activity and noise) to show equal or better spatial tuning for visual and motor codes.

Figure 9 shows the results of this analysis for visual (Fig. 9*A*) and motor (Fig. 9*B*) responses, showing the distribution of TG fits for the original MU population along the *x*-axes, the distribution for the reconstructed population along the *y*-axes, and site-matched (visual, *n* = 88; motor, *n* = 90) data in the intervening scatterplots. Comparing the distributions, although the reconstructed activity still showed a general trend to shift from T toward G in the motor code (Fig. 9*B*), the distributions of the reconstructed codes (*y*-axes) were broadly dispersed and lacked the clear unimodal peaks found in the original MU dataset (*x*-axes). Briefly, untuned and other neurons do not omit the original MU signals. Furthermore, no correlation existed between the best fit scores for site-matched recombined versus original analysis for both the visual (Spearman *R* = −0.05; slope = −0.23 ± 0.18; *p* = 0.63) and motor (Spearman *R* = 0.02; slope = 0.11 ± 0.20; *p* = 0.82) populations. In part, this may be because only some of the spatially tuned MU sites yielded spatially tuned reconstructed datasets [visual, 62/88 (70%); visual and motor, 66/90 (73%)]. Again, the residuals were significantly higher (Mann–Whitney *U* test; *p* < 0.001) for the original MU signal than the reconstructed MU data for both the visual and motor responses, further suggesting that the MU signal is not a simple sum of several SUs.

In summary, these analyses show that MU activity reconstructed from SU data does not replicate, let alone improve, the coding schemes in the original MU activity. This suggests that there is useful information in the original MU dataset that is not simply the sum of the “best” neurons but also depends on background noise and baseline signals that are removed in the process of spike sorting for SU analysis.

### Regular versus fast spike SUs: TG continuum analysis

**Figure 10.**
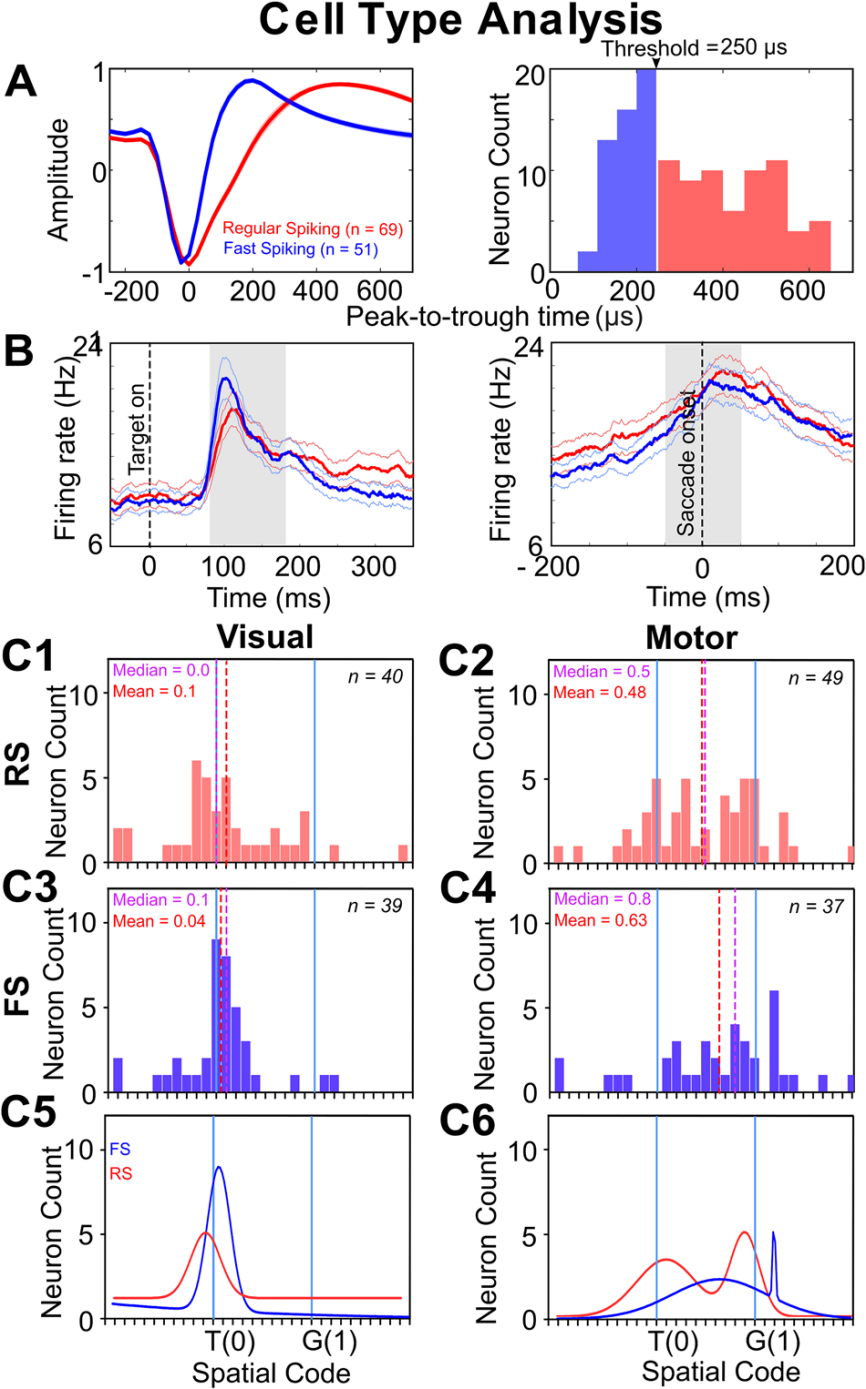
Biophysical classification of cell types. ***A***, Left, Spike waveforms (mean ± SEM) of two types of neurons dissociated based on trough-to-peak time: regular-spiking (RS, red) and fast-spiking (FS, blue). Right, Distribution histogram of RS and FS cells with a cutoff threshold of 250 µs. ***B***, Overlapped average spike density plots (95% confidence) for visual (left, aligned to target onset) and motor responses (right, aligned to saccade onset) of RS (red) and FS (blue) neurons. Gray shaded area represents the sampling window. ***C1***, Frequency distribution of best fits of spatially tuned RS visual responses (*n* = 40), with the best fit closer to T (mean = 0.1; median = 0.0). ***C2***, Frequency distribution of best fits of spatially tuned RU motor responses (*n* = 49) with the best fit in between T and G (mean = 0.48; median = 0.5). ***C3***, Frequency distribution of best fits of spatially tuned FS visual responses (*n* = 39) with a best fit closer to T (mean = 0.04; median = 0.1). ***C4***, Frequency distribution of best fits of spatially tuned FS motor responses (*n* = 37) with a significantly shifted distribution toward G (mean = 0.63; median = 0.8). ***C5***, The sum of two Gaussian distributions of RS (red) and FS (blue) visual responses (only one peak closer to T). ***C6***, The sum of two Gaussian distributions of RS (red) and FS (blue) motor responses (bimodal distribution in both cases). See Extended Data Figure 10-1 for more details.

We next tested the contributions of different SU cell types to our FEF visual and motor population codes, specifically “regular spiking” cells (RS, putatively linked to pyramidal neurons) versus “fast-spiking” cells (FS, putatively linked to inhibitory interneurons; Niell and Stryker, 2010; Ding and Gold, 2012; Vinck et al., 2013; Chanauria et al., 2016; Thiele et al., 2016; Dasilva et al., 2019; Trainito et al., 2019). To do this, we categorized our SUs based on their spike width duration (peak-to-trough time), with a split threshold of 250 µs: neurons with spike width >250 µs (Vinck et al., 2013; Thiele et al., 2016; Dasilva et al., 2019) were labeled as regular spiking (RS, *n* = 69) and neurons with ≤250 µs were classified as fast spiking (FS, *n* = 51) neurons (Fig. 10*A*). We also identified and analyzed 27 triphasic neurons (Suárez-Solá et al., 2009; Barry, 2015). These data are shown in Extended Data Figure 10-1 but we considered the population distribution to be too small and discontinuous to draw firm conclusions.

10.1523/ENEURO.0413-23.2024.f10-1 Figure 10-1 **Triphasic waveforms**. **A1,** Frequency distribution of best fits of spatially tuned visual responses (n = 17, mean = 0.41; median = 0.2), significantly shifted (p = 0.011, one-sampled Wilcoxon signed rank test). **A2**, Frequency distribution of best fits of spatially tuned motor responses (n = 17, mean = 0.7; median = 0.8), significantly shifted from T toward G (p = 0.0003, one-sampled Wilcoxon signed rank test). **B1-B2**, The sum of two Gaussian distributions for visual (**B1**) and motor (**B2**) responses (bimodal distribution in both cases). **C,** Spatiotemporal progression for triphasic neurons (n = 27), characterized by several dips and rises from visual to motor responses. The solid circle represents a significant shift (p < 0.05, one-sampled Wilcoxon signed rank test) from T toward G. Download Figure 10-1, TIF file.

Figure 10*B* shows the superimposed mean (and 95% confidence interval) spike density plots of the RS (red) and FS (blue) neurons for the visual (left) and motor responses (right). As reported previously (Constantinidis and Goldman-Rakic, 2003; Trainito et al., 2019), the firing rate for the FS neurons was slightly higher during the visual response, i.e., the mean of blue curve is above the red curve in our sampling window (gray area).

Figure 10, *C1* and *C2*, shows the distribution of visual (C1) and motor (C2) responses for RS neurons. Overall, the visual distribution (Fig. 10*C1*) fit closer to T (*n* = 40; mean = 0.1; median = 0.0) with no significant shift from T (*p* = 0.52; one-sample Wilcoxon signed rank test) or any secondary peak. Like the SU population, RS motor distribution had a primary peak near G, with a secondary peak near T. Seventy-five percent of RS visual responses showed considerable target retention with best fit scores below 0.5 (closer to T than G). Conversely, 49% of motor responses showed target retention, with best fit scores below 0.5 (closer to T than G). Overall, the RS motor distribution (*n* = 49; Fig. 10*C2*) was significantly different from the visual distribution (*p* = 0.002; Mann–Whitney *U* test) and significantly shifted from both T and G toward an intermediate best fit point between T and G (mean = 0.48; median = 0.5; *p* < 0.0001; one-sample Wilcoxon signed rank test).

Figure 10, *C3* and *C4*, shows the same analysis for the FS neurons. Like RS neurons, the visual distribution (Fig. 10*C3*) fit closer to T (*n* = 40; mean = 0.04; median = 0.1) with no significant shift from T (*p* = 0.40; one-sample Wilcoxon signed rank test) and the motor distribution (Fig. 10*C4*) significantly shifted toward G (mean = 0.63; median = 0.8; *p* < 0.0001; one-sample Wilcoxon signed rank test). Again, the FS visual distribution was significantly different from the corresponding motor distribution (*p* < 0.0001; Mann–Whitney *U* test). Ninety-two percent of FS visual responses showed considerable target retention with best fit scores below 0.5 (closer to T than G). On the other hand, 32.4% of motor responses showed target retention, with best fit scores below 0.5 (closer to T than G).

Figure 10, *C5* and *C6*, superimposes the RS and FS Gaussian distributions for the visual and motor fit frequency distributions shown above. Overall, there was no significant difference between the RS and FS visual (*p* = 0.98; Mann–Whitney *U* test) and motor distributions (*p* = 0.35; Mann–Whitney *U* test) but see the delay response below.

### Spatiotemporal analysis: MU versus SU populations

**Figure 11.**
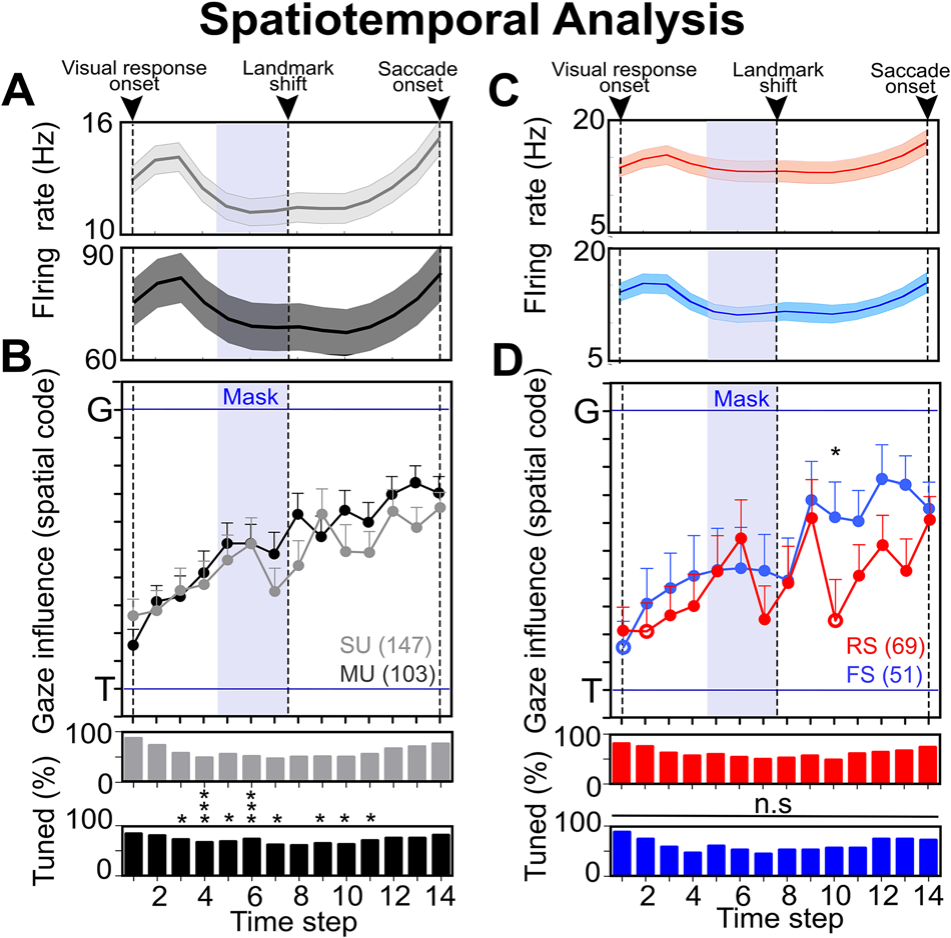
Spatiotemporal analysis at the population level. ***A***, Time-normalized spike density plot for the SU (top) and MU (bottom) populations. The arrows at the top indicate major time events in the task. ***B***, Top, Progression of the spatial code (gaze influence, mean ± SEM) along the T–G continuum with time (at different time steps) from visual response onset until the saccade onset. There was a gradual and significant progression toward G for both SU (gray) and MU (black) activity, with no significant difference between either. Bottom, Percentage of spatially tuned neurons at each time step for SU and MU data. There was a significant difference between the proportions (*p* < 0.05; *z*-test for proportions) at several steps during the delay period. ***C***, Time-normalized spike density plot for the RS (red) and FS (blue) neurons. ***D***, Progression of the spatial code mean ± SEM) along the T–G continuum from visual response onset until the saccade onset. There was a gradual and significant progression toward G for both RS and FS neurons. Notably, there was a gradual progression in both cases until step 6, but then the RS neurons displayed a few dips and rises until the saccade onset. However, FS neurons showed a sudden shift at step 8 that is mostly maintained until saccade onset. In the end, both curves merged at the same point. Postlandmark shift, there was a visual divide between the RS and FS spatiotemporal progression, with a significant difference at step 10 (*p* = 0.03; Mann–Whitney *U* test). Bottom, Percentage of spatially tuned neurons at each time step for RS and FS neurons. No significant difference was observed between the proportions (*p* > 0.05; *z*-test for proportions) of significantly tuned data between both classes. The blue shaded area represents the duration of the mask. See Extended Data Figure 11-1 for more details.

Finally, we performed the 14-step time-normalized analysis (Bharmauria et al., 2020) from visual response until the saccade onset (Fig. 11) for both SU and MU activity (Fig. 11*A*,*B*) and different cell types (Fig. 11*C*,*D*). This procedure (see Materials and Methods for details) permitted us to track the T–G progression along the spatiotemporal domain. Figure 11*A* represents the normalized spike density plots of the SU (top) and MU (bottom) data: the initial peak indicates visual activity (in response to the target), and then activity diminishes during the memory delay, to then rise again when the saccade was just imminent.

10.1523/ENEURO.0413-23.2024.f11-1 Figure 11-1 **A**, Spatiotemporal progression for untuned neurons / sites (n = 48 / 31). No clear trend was observed for the SUs (light green) but the corresponding sites (dark green) showed a weak signature of T-G progression. **B**, Spatiotemporal progression for tuned + untuned neurons / sites (n = 134 / 195). A similar progression to the tuned only neurons / sites (Fig. 10), but untuned neurons seem to dilute the TG progression (significant difference between SU and MU at time steps 7, 8, and 11; p < 0.05, Mann-Whitney U test). The solid circle represents a significant shift (p < 0.05, one-sampled Wilcoxon signed rank test) from T toward G. At all steps (**A** and **B**), the corresponding proportions of spatially tuned data were significantly different from each other. Download Figure 11-1, TIF file.

Figure 11*B* (top) shows the visuomotor transformation between the SU and MU activity: the gray curve represents the SU transformation along time (Bharmauria et al., 2020), whereas the black curve corresponds to the visuomotor transformation in the MU activity. Although subtle differences between the SU and MU data are evident at certain steps between SU and MU, there was no significant difference between the curves (*p* > 0.05; Mann–Whitney *U* test). In both cases, there was a gradual transition from T–G, however, in the case of the MU activity, the transition appears to be “smoother” than the SU, without the dips and rises seen during and after visual mask presentation. Specifically, in the SU activity, the code moves back and forth several times after time step 6, but in the MU activity, the code is mostly maintained from time step 8 (postlandmark shift) until the 14th step, when the gaze saccade was just imminent. Figure 11*B* (bottom) shows the proportion of significantly tuned neurons along the spatiotemporal domain in the SU and MU population. At several steps (3, 4, 5, 6, 7, 9, 10, and 11), the proportion of significantly tuned MU sites was significantly higher (*p* < 0.05; *z*-test for proportions) than the SU data.

In contrast, the untuned populations showed a highly variable spatiotemporal progression, with the untuned SU population showing no discernable pattern through time, and the MU population showing (at best) a weak visual-to-motor trend (Extended Data Fig. 11-1*A*). When we pooled the untuned SU data with the tuned SU data and the untuned MU with the tuned MU data (Extended Data Fig. 11-1), the general trend of the original spatially tuned data remained unchanged, but with significant fluctuations in MU steps 7, 8, and 11 (*p* < 0.05; Mann–Whitney *U* test). Overall, this tended to confirm that the original spatially tuned and MU datasets were superior.

### Spatiotemporal analysis: regular versus fast spiking cells

Finally, we asked if different cell types (i.e., RS and FS) might contribute differently to the spatiotemporal transformation, particularly during the delay period. When we performed our spatiotemporal analysis on the RS and FS SU populations (Fig. 11*C*,*D*), they showed a similar T–G progression until step 6 but then showed opposite trends during the late delay period. Specifically, FS neurons showed a sudden and sustained shift toward G after the mask offset (coinciding with landmark shift), whereas the RS curve (after some transient variations) remained lower, i.e., closer to T until the curves rejoined near G during the motor response (corroborating the analysis in Fig. 10). The difference between these two curves was significant at step 10 (*p* = 0.03; Mann–Whitney *U* test).

## Discussion

The aim of this study was to compare sensorimotor transformations derived from SU activity versus MU activity from the same FEF recording sessions, using data from a signal-rich memory-delay gaze task. Our analysis of visual and motor response fields (Figs. 4–6) results indicated that both SU and MU activity show a fundamental progression from target coding in the visual response to gaze coding in the motor response, except that MU activity does not reveal secondary characteristics, i.e., motor prediction in the sensory response and target location retention in the motor response (Fig. 7). This basic transformation was also evident within single SU/MU recording sites that showed both visual and motor activity (Fig. 8) but was absent when MU populations were reconstructed from SU activity (Fig. 9). Further, SU and MU activity showed a similar temporal target-to-gaze progression during the memory delay, although the transition in MU activity was somewhat “smoother” (Fig. 11). Finally, when SUs were sorted into RS versus FS cells, they showed similar visual and motor codes (Fig. 10) but their spatiotemporal progression transiently diverged during the late planning phase (Fig. 11). One might conclude that MU activity provides an accurate measure of the overall sensorimotor transformation in our dataset (in some ways more clearly than SU activity), whereas SU activity contains additional information that might prove important for more cognitive aspects of the task.

### Multiunit versus single-unit activity

Recently, there has been increased interest in comparing SU and MU activity in both animals and humans (Burns et al., 2010; Christie et al., 2015; Gilja et al., 2015; Pandarinath et al., 2017; Drebitz et al., 2019; Trautmann et al., 2019; Ahmadi et al., 2021; Ayar et al., 2023). For example, Trautmann et al. (2019) suggested that MU recordings provide both the practical benefit eliminating spike sorting (required for SU analysis) and reducing the dimensionality of the signal without losing important information content. They performed this analysis on three sets of monkey data from different labs (Churchland et al., 2012; Ames et al., 2014; Kaufman et al., 2014) and reached the same conclusion. This fits with the theory of random projections from high-dimensional statistics (Indyk and Motwani, 1998; Dasgupta and Gupta, 2003; Ganguli and Sompolinsky, 2012; Lahiri et al., 2016), i.e., to recover the geometrical properties of a low-dimensional manifold embedded in a high-dimensional space, one can bypass the coordinates of the high-dimensional space. The results of our study tend to agree with this conclusion, with certain caveats that we will discuss in more detail below.

### Physiological interpretation of MU response fields

Technically, the MU response fields that we used in our model fits arise from a conglomeration of cellular activities weighted according to their relative firing frequency, strength, and distance from the tip of each electrode (Bullock, 1997; Buzsáki, 2004; Quian Quiroga and Panzeri, 2009; Burns et al., 2010; Mattia et al., 2010; Land et al., 2013; Rey et al., 2015). However, this could be interpreted physiologically as being like the response field of a hypothetical downstream “neuron” that receives inputs from the same neurons with corresponding synaptic weightings. Our control analysis of reconstructed MU activity containing SUs from the same sites shows that MU response fields are not simply the sum of response fields obtained from spike sorting. Perhaps, surprisingly our model fits to these reconstructed populations were worse, in the sense that coding along the TG continuum was dispersed and there were no correlations with the original MU TG codes, while some sites lost their spatial tuning (Fig. 9). This shows that the data missing from the reconstructed MUs (“small” signals, untuned signals, multiple baselines, and noise) somehow made a meaningful contribution to the overall MU code. This finding is analogous to the phenomenon that population decoding is more successful when untuned neurons are included in the analysis (Zylberberg, 2018; Pruszynski and Zylberberg, 2019; Levy et al., 2020).

### Implications for sensorimotor control

As noted in the introduction, oculomotor scientists have spent many years studying the transformation from visual target coding to the coding of saccade metrics in saccades and head-unrestrained gaze shifts (Bruce and Goldberg, 1985; Hanes et al., 1995; Everling and Munoz, 2000; Umeno and Goldberg, 2001; Gandhi and Katnani, 2011; Sadeh et al., 2015; Sajad et al., 2015; Schall, 2015). Visual and motor tuning have been dissociated by introducing various position or memory-related errors (Mays and Sparks, 1980; Gnadt et al., 1991; White et al., 1994; Umeno and Goldberg, 2001; Masselink and Lappe, 2021; Heusser et al., 2022), training animals to make “antisaccades” opposite to the stimulus (Everling and Munoz, 2000), or introducing an intervening saccade (Baizer and Bender, 1989; Camalier et al., 2007). Some of these approaches introduce top-down signals that might alter the circuitry involved (Gaymard et al., 1998; Munoz, 2002; Pierrot-Deseilligny et al., 2002; Munoz and Everling, 2004), but more recent model-fitting analyses confirm a transition from target-to-saccade coding in SC (Sadeh et al., 2015, 2018), FEF (Sajad et al., 2015, 2016; Bharmauria et al., 2020), and SEF (Bharmauria et al., 2021) activity, even in reactive gaze shifts to visual targets (Sadeh et al., 2020).

The current study shows that both SU and MU activity retain this target-to-gaze transformation, even in the presence of a relatively complex stimulus array. A previous analysis of the same dataset showed that some visual responses also encoded landmark locations (Schütz et al., 2023) and the motor response was modulated by landmark shifts (Bharmauria et al., 2020) but overall, Te to Ge was retained (Bharmauria et al., 2020). Here, we showed that MU activity shows the same sensorimotor transformation (in some ways “cleaner”, providing a “purer” measure of target and gaze coding at the population levels). Further, whereas the spatiotemporal progression of the SU data showed various dips and rises during the delay period, perhaps related to microsaccades (Heusser et al., 2022), the MU data showed a “smoother” transition, suggesting a more robust sensorimotor signature. Considering the relative ease of recording online MU activity (without the need for off-line spike sorting), this suggests that MU activity could be sufficient for certain practical applications requiring low-dimensional information (Trautmann et al., 2019; Ayar et al., 2023). For example, MU activity could be observed to rapidly assess the health of sensorimotor transformations during presurgical recordings in patient populations.

### Implications for the cognitive aspects of movement control

Higher dimensional neural data from single electrodes may require pooling across several simultaneously recorded MU channels to confidently estimate the neuronal population dynamics (Gao and Ganguli, 2015; Trautmann et al., 2019). Here, the secondary Ge code that was observed in some SU visual responses and the secondary Te code that was observed in some motor responses (Bharmauria et al., 2020) was attenuated for MU analysis. Specifically, these secondary codes are dominated by the primary codes in population statistics (Fig. 4), and likewise, they appeared to be “drowned out” by the primary codes of most neurons in the MU activity. Ge activity in the visual response could reflect internal noise that ends up influencing variable errors in final gaze position (Sajad et al., 2015, 2016, 2020) but it has been suggested that the prefrontal cortex is involved in the intentional prediction of future gaze errors (Fu et al., 2023). Conversely, the retention of Te in the motor response could be used for motor learning, updating perception, and/or corrective saccades (Shadmehr and Mussa-Ivaldi, 1994; Wolpert et al., 2011; Dash et al., 2015; Mohsenzadeh et al., 2016; Stavisky et al., 2017; Lundqvist et al., 2018; Sajad et al., 2020; Mathew and Crevecoeur, 2021; Wagner et al., 2021).

### Conceptual model

**Figure 12.**
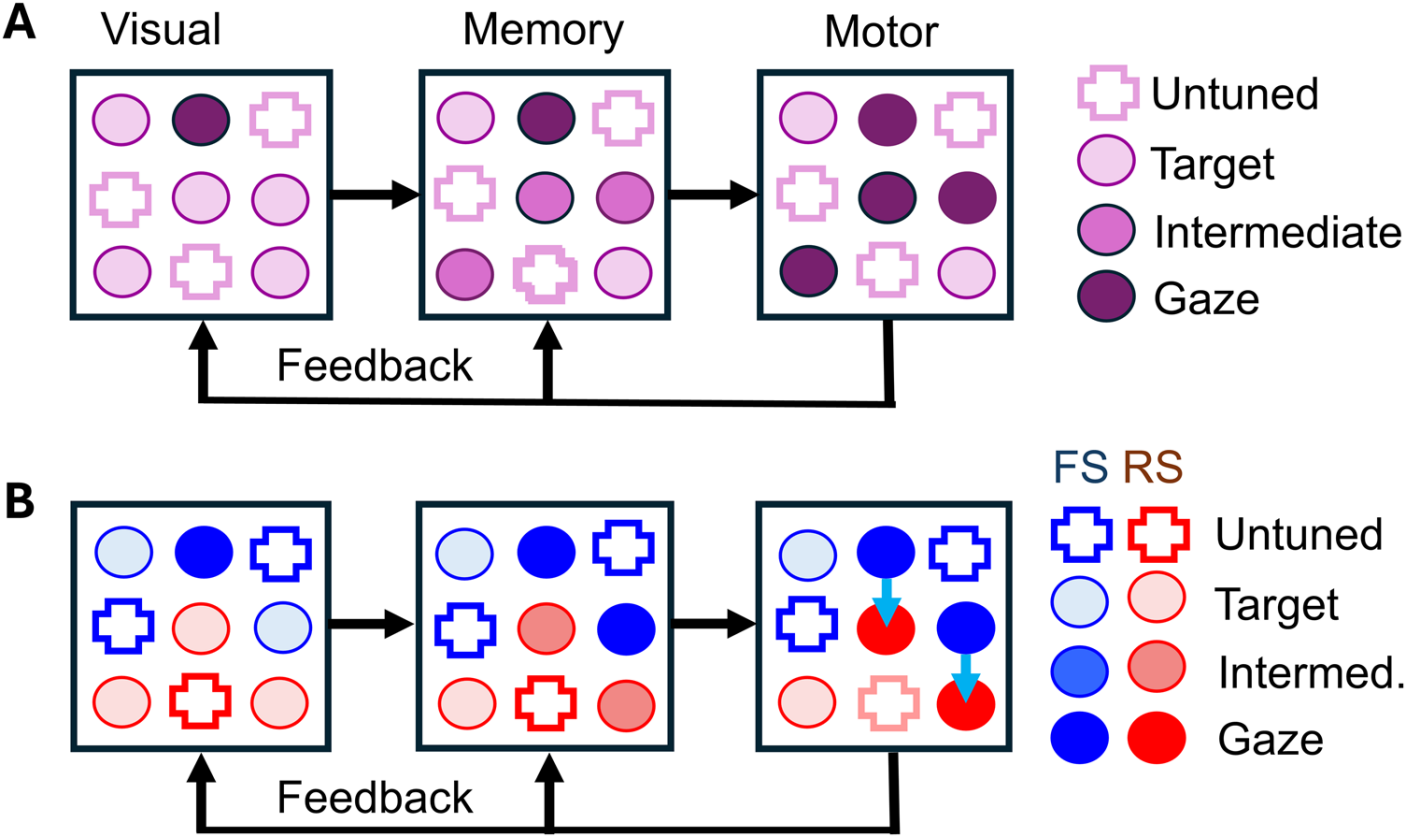
***A***, Neural circuit model for SU activity in our task. During the sensory response, neurons mostly encode the target-in-eye coordinates (T; light circles) but some code gaze-in-eye coordinates (G; dark circles) or are spatially untuned. The sensory response passes information to delay circuits involved in retention of visual information and planning signals (intermediate and G codes). During the motor response, neurons primarily encode G, but some retain T information (see text for details). ***B***, Same circuit broken down into fast spiking (FS; blue) and regular spiking (RS; red) neurons. This circuitry partakes in the same T–G transformation as part A, with two differences: FS cells code relatively more G activity during the delay period, and this information appears to be passed (cyan arrows) to RS units in the motor response.

Figure 12*A* shows a schematic circuit that could explain how sensory, cognitive, and motor responses arose in our data, breaking the process down to cell activity during the visual response, delay activity (memory and planning signals), and the motor response. Visual input to the sensory circuit leads to predominantly eye-centered spatial tuning (light circles; Mullette-Gillman et al., 2005; Snyder, 2005; Sajad et al., 2020), but some neurons (dark purple circles) already predict future gaze (Ge). Other untuned neurons (crosses) may contribute to population dynamics, as suggested by our MU analysis and previous decoding experiments (Bharmauria et al., 2016a; Zylberberg, 2018; Pruszynski and Zylberberg, 2019; Levy et al., 2020). The sensory circuit relays information to the delay circuit, which continuously retains and updates signals for T, G, and intermediate T–G codes involved in target memory retention and planning the sensorimotor transformation (Sajad et al., 2015, 2020; Bharmauria et al., 2020). This circuit likely provides feedback to the sensory circuit (Franklin et al., 2012; Chatham and Badre, 2015; Sajad et al., 2020), involving LIP, dorsolateral prefrontal cortex, and FEF (Christophel et al., 2017; Lundqvist et al., 2018; Pinotsis et al., 2019). As noted above, some of these signals are likely obscured in the MU response (as it averages the contribution of all neurons in the circuit), resulting in a “smoother” T–G transition (Fig. 11*A*).

Lastly, when a gaze shift is cued, activity from the memory circuit is transferred to the motor circuits (blue) in a memory–motor transformation (Bharmauria et al., 2020). This has been shown to involve an additional population shift toward Ge coding (Sajad et al., 2016). Differences of Ge from Te may result from internal noise, endogenous inputs (top-down, updating), or exogenous influences from the internal world (Bharmauria et al., 2020; Abedi Khoozani et al., 2022; Schütz et al., 2023). The predominant dark purple neurons produce the most accurate Ge command and thus potentiate the gaze shift, but other neurons remain untuned or code Te signals, providing the system with an untainted visual memory signal for learning or use in subsequent saccades (Barth and Poulet, 2012; Bharmauria et al., 2016a; Zylberberg, 2018; Pruszynski and Zylberberg, 2019; Levy et al., 2020). Overall, the ensemble code shows a progressive transition from Te to Ge across the visual–memory–motor circuitry (Sajad et al., 2020). Once again, the other secondary codes are attenuated in MU activity, with the result that a similar drawing of the MU circuit would look more like a simple transition from light purple (target coding) to dark purple (gaze coding) while missing the various nuances.

### Role of regular-spiking (RS) and fast-spiking (FS) Neurons

Figure 12*B* further breaks down the delay activity into RS neurons (red) and FS neurons (blue) neurons. Our results show that both neuron types mostly encode target location (light circles) in the visual response and future gaze position (dark circles) in the motor response (Fig. 10). However, in the late delay period, FS neurons predominantly code gaze whereas RS neurons maintain intermediate codes closer to T (Fig. 11). This suggests that FS (inhibitory interneurons) are not just involved in memory, but also in motor planning, and that these gaze planning signals may be integrated into the RS (pyramidal) cells at the last moment (see arrows in the motor circuit). This gaze signal would then be output to other brain areas, like the superior colliculus and brainstem burst neurons (Scudder et al., 2002; Gandhi and Katnani, 2011).

### General implications for population coding

Neural data is fundamentally noisy, arising both from endogenous biological noise (Faisal et al., 2008) and from methodological constraints. In the cortex, neurons fire sparsely and exhibit emergent properties in relation to a task/stimulus, but not all activated neurons are necessarily part of the emergent network (Buzsáki, 2010; Barth and Poulet, 2012; Singer, 2013; Bharmauria et al., 2016a; Molotchnikoff et al., 2019; Levy et al., 2020). However, if a neural population has homogenous properties, i.e., similar tuning curves, averaging the ensemble activity of several neurons might provide a better estimate of the network's goal (Quian Quiroga and Panzeri, 2009; Trautmann et al., 2019). Thus, this level of analyses may work best for brain areas with homogeneous populations (Panzeri et al., 2015). This suggests some degree of fundamental homogeneity in the signals we recorded here, despite the relative lack of topography in the recording sites. Moreover, it may also provide information about network level changes after brain damage (Chaudhary et al., 2016) or neuroplasticity (Kohn, 2007; Bharmauria et al., 2022).

Even in the presence of dissimilar tuning curves, or underlying decorrelation, one might still accurately estimate neural manifold geometries using MU channels (Quian Quiroga and Panzeri, 2009; Panzeri et al., 2022). For example, the response field of the motor neuron in Figure 6*C* has a hotspot in the third quadrant, but the second neuron (data not shown) recorded from the same site had a hotspot in the second quadrant. Despite this, when their MU activity was analyzed, it was possible to create a population hotspot and determine its motor code (Ge). Consistent with our findings, it has been suggested that only extreme anticorrelations between neural tuning curves disrupt accurate estimation of population manifolds (Trautmann et al., 2019).

### Conclusion

This study's aim was to determine what information is retained and lost in the methodological transition from SU to MU analysis. An advantage here is that we were able to combine a well-defined sensorimotor system, task, and analytic technique to compare signal coding across both situations. Overall, we found that FEF MU activity carries an excellent representation of the overall sensorimotor transformation for memory-guided gaze shifts but loses some nuances that may be important for the more cell-specific cognitive and planning aspects of the task. We conclude that MU activity has advantages for applications that must prioritize time over sophistication (potentially including presurgical recordings and some brain-machine interfaces), whereas SU remains relevant for tasks and analyses where more subtle secondary signals and modulations are important.

## Synthesis

Reviewing Editor: David Schoppik, New York University Decisions are customarily a result of the Reviewing Editor and the peer reviewers coming together and discussing their recommendations until a consensus is reached. When revisions are invited, a fact-based synthesis statement explaining their decision and outlining what is needed to prepare a revision will be listed below. The following reviewer(s) agreed to reveal their identity: Marc Sommer, Nabil Daddaoua.

I think that in the limited scope of the aims - examining how signals in SU and MU activity differ in a well-established task - the report makes a useful contribution to the field. The report did not include much attempt to explain the differences, however, either analytically or in terms of scholarly discussion. The results are not particularly surprising, given that MU activity is essentially a "messier" signal than SU activity and likely includes a lot of neurons that would be eliminated in initial screening of SU neurons (e.g. non-task related neurons). Hence the loss of subtle signals in MU vs SU activity, and the smoother transition in coordinate frame representations, are to be expected. Sampling biases of singly isolated neurons relative to MU activity may be a confound as well, but is not addressed. Overall, the results may be useful to the community of scientists who want or need to collect primarily MU activity (such as BMI researchers), but the manuscript lacks depth of analysis and discussion that could benefit future studies of the topic.

Major comments:

1. The constituent signals in MU activity vs. the SU sample: What is MU activity? It is the summed extracellular potential of an unknown number of single neurons from an unknown volume of tissue. What is SU activity, in this report? It is well-isolated single neuron activity that is pre-screened to eliminate units that were un-interesting or irrelevant to the task and analyses. The screening was fairly extensive as described in lines 277-281. It removed 53% of the 312 SU neurons recorded in the task (leaving 147 to analyze). The MU activity was pre-screened the same way, but must have consisted of many neurons that, individually, would not have passed the screening criteria. Hence the MU activity was "diluted" by signals that would not have been considered acceptable for SU analyses.

For the practical purpose of describing the signals in the MU activity, this may be okay. MU activity is easy to record, and sometimes it is the only activity possible to record (e.g. if using fixed arrays as is often the case for BMI). The results, as presented, could be useful for an important but limited audience.

For the broader audience of neuroscientists, however, the manuscript falls short in its analyses. A more apples-to-apples comparison of MU and SU activity could be accomplished, we think, which would have more impact. This could involve constructing simulated MU activity by summing the signals of random subsets of SU neurons (using all 312 of them, not just the ones passing the inclusion criteria). Would that summation explain the results found for the MU activity, e.g. the loss of signal bimodality (Figure 7) or the smoother T-G transition (Figure 9)? If so, how many of the SU neurons need to be summed to achieve similarity with MU activity? That could give one an estimate of the actual numbers of neurons contributing to the MU samples. One could imagine a lot of analyses like this, based on unscreened SU neurons to simulate MU activity, that could be very illuminating to the field. It's a unique, beautiful MU and SU dataset, expertly collected from the same sites, monkeys, and tasks, that presents a valuable but missed opportunity for higher impact with just some further analyses.

2. Potential sample bias in SU signals vs. MU aggregate signals: Single-unit recording is well known to be affected by sample biases. As one example, neurons with larger extracellular potentials are easier to isolate, which means that larger pyramidal neurons may be over-represented (e.g. Towe &Harding 1970, Exp. Neurol. 29: 366-381). Investigator biases can occur as well, such as focusing on fast firing rate neurons or neurons that seem especially task-related. Sampling biases may also affect MU recordings, although less is known about that. Potential sampling biases are not addressed at all in the manuscript, but it seems possible that they may contribute to the differences found between SU and MU samples.

We also note that several papers have evaluated the putative types of neurons that can be recorded as SU activity in the FEF, based on action potential waveform shapes and other criteria (Ding &Gold 2012, Cerebral cortex, 22: 1052-1067; Shin &Sommer 2012, J Neurophysiol. 108: 2144-2159; Thiele et al. 2016, J Neurosci., 36: 7601-7612; Trainito et al. 2019, Current Biology, 29: 2973-2982; Dasilva et al. 2019, PNAS, 116: 20180-20189). One might consider applying that sort of approach to evaluate whether the SU samples of this report seem biased toward pyramidal neurons or not. If a reasonably uniform distribution of putative pyramidal and inhibitory neurons is found, it could provide an argument that the SU samples are similar to the MU samples in terms of neural types. Or, if the SU sample seems biased toward one type of neuron or another, the investigators could leverage that information to adjust for sampling bias if they model the MU activity using the entire SU dataset as suggested in point 1 above.

3. Bottom line: The main conclusion of the manuscript is that task-related signals in MU activity are more homogeneous than those in SU activity, but that is not particularly surprising. MU activity was screened for inclusion but must have contained many individual neurons that would fail the screening, and yet that MU activity was compared only to SU neurons that passed the screening. It is clear from the manuscript that the investigators are experts in modeling and neurophysiology. The manuscript would benefit from application of that expertise to a quantitative explanation of MU signal homogeneity (as suggested in points 1 and 2 above), or by a more scholarly discussion that carefully addresses the possible reasons for discrepancies in SU and MU signal content, or both. The Discussion Figure 10 and its associated text addressed the SU activity in depth, but the physiological factors that could explain the MU findings were not addressed with comparable clarity and detail.

Minor comments:

Lines 70-71: "Further, MU activity profiles are similar to local field potential (LFP) signals but tend to show less correlation between nearby neurons..." Do the authors mean "sites" instead of "neurons" at the end? Each MU activity profile is from a lot of neurons. Or do the authors mean correlation between neurons extracted (sorted) from a single MU recording? Could be clarified.

Line 147: Possible confusing typo, maybe "coils" instead of "cells"?

Lines 241-243 and elsewhere: "Figure 2C-D displays the overlapped and averaged spike density plots for top 10 % (for each neuron and MU site) trials for the SU and MU." We found it confusing why 90% of trials were omitted from analysis, until we realized that this is how to isolate trials from the hot spot of the receptive or movement field given the mapping techniques used. It will help the readers a lot if the authors explain the criteria and rationale for picking the top 10% of trials, particularly since the manuscript should not assume that readers are familiar with previous papers with respect to this fundamental step in the analyses.

Lines 269-270: "As the MU cluster comprises different spikes, it generally has a lower amplitude than the individual spikes sorted from it." This seems like an overly simplistic, and potentially misleading, explanation. It's more that the electrode isn't immediately near any one neuron, so one collects more signals but they are all further away than for an SU signal (assuming the same density of neurons for MU and SU recordings). We suggest rewording this explanation, particularly explaining the following: what do you mean by cluster, is it the nearby tissue at the recording site? When the authors write amplitude do they mean the resulting amplitude after applying the single unit threshold to the multi-unit data? Please reformulate this sentence for clarity.

Lines 270-272: "We re-thresholded the high-pass filtered raw data to isolate the MU activity from the 40 kHz signal with a spike detection threshold of 3.5 standard deviations (SDs). What 40 kHz signal? We don't see that explained anywhere. And 3.5 SDs of what? The noise level? For replication and reproducibility, this method should be explained better.

Line 273: "[the procedure described in point 5 above] led us to spiking activity recorded from several neurons recorded within a vicinity of ∼100-200 μm around the electrode tip." It seems odd to state that estimated radius so matter-of-factly. Perhaps this could be qualified a bit, perhaps like "According to previous studies, the neurons were likely ~100-200 um from the electrode tip, compared to ~X um from single neurons in the SU recordings" (and fill in the X, it would be useful for readers to understand that comparison).

Figure 3 legend: L and L' abbreviations are defined but not used in the figure anywhere we could see.

Line 336: "Sum" of Squares, not "Some."

Lines 370-372 and elsewhere: "The spatial continuum between Te and Ge ('T-G continuum') provided the best overall description of the data..." Here, and later in the paper up until Figure 7, the continuum is described as ranging from Te to Ge. But in Figure 7 many values are shown <Te and >Ge. This is a confusing disconnect between the text and that Figure. Please clarify the text in a way that readers aren't surprised by the range of values displayed in Figure 7.

Line 395: "Thus, we removed the time steps..." Please provide a quantification on how many time steps were removed, on average, per session.

Lines 398-400: "CI for an SU / MU site, ..." is not a complete sentence.

Figure 4B and 4D: The p-value for dE was also very close to 1, it appears. Numerically, Ge wins, but it seems odd to not even mention that dE is nearly equally well fitted.

Line 406-407: "To accommodate this variable trial-time for transformations between these events, we time-normalized the duration of each trial." It would help the reader if that sentence were followed with something like, "This normalization was conducted only for the Spatiotemporal Analysis presented at the end of Results." We were confused throughout the Results, expecting to see the normalizations, until we got to the final section where it was used for Figure 9. Please rewrite.

Lines 498 and 531: Would help the reader to reword these opening sentences as "shows the visual response field" and "shows the motor response field," respectively.

Line 567: "...peaked in different quadrants upper-left......upper-right...." Should read: peaked in different quadrants lower-left......upper-left.

Line 569: "(the second neuron, not shown, ...)". Do the authors mean "a" second neuron? We don't see anywhere else that it was mentioned that two SUs were isolated from this recording.

Line 570: The authors write: ... second quadrant. The authors never defined a ranking order for quadrants (first vs second etc..). Please clarify.

Line 627: "one sampled Wilcoxon signed rank test" - against what theoretical value?

Line 628: Fig. 8B2 should read: 7B2.

Line 712: "... much smoother..." Please provide a quantification of this effect. One might just compare the sum of the unsigned differences between 2 successive data points.

Line 870: Figure 7C, must read Figure 6C.

## Author Response

We thank the reviewers for their positive comments on the manuscript. We have addressed these comments as follows. Please note that the line numbers below correspond to the marked version of the manuscript.

Major comments:

1. The constituent signals in MU activity vs. the SU sample: What is MU activity? It is the summed extracellular potential of an unknown number of single neurons from an unknown volume of tissue. What is SU activity, in this report? It is well-isolated single neuron activity that is pre-screened to eliminate units that were un-interesting or irrelevant to the task and analyses. The screening was fairly extensive as described in lines 277-281. It removed 53% of the 312 SU neurons recorded in the task (leaving 147 to analyze). The MU activity was pre-screened the same way, but must have consisted of many neurons that, individually, would not have passed the screening criteria. Hence the MU activity was "diluted" by signals that would not have been considered acceptable for SU analyses.

REPLY: We agree: any analysis of unsorted multiunit activity contains a 'grab bag' of different units, some of which might be excluded in SU analysis based on factors such as good isolation and (in our case) significant spatial tuning. This is exactly the question posed in this study: what is the same and what is different when we apply the same analysis criteria to SU vs. MU activity? We find that some signals are retained in the MU analysis, and some are lost, leading to the main conclusions of the study. However, we 2 agree that it is also important to control for sampling bias and understand the reasons for SU/MU differences (see responses to major comments below).

ACTION: We have made these points more explicit in the introduction (page 3, lines 52-53), and we have posed the new question in the introduction, i.e., what causes the differences (page 3, lines 52-53). Also, in response to the more specific comments provided below, we have further performed several additional analyses on neurons, including an analysis of single unit populations that includes untuned neurons (Extended Data Figure 11-1), an analysis of reconstructed multiunit populations (new Figure 9), and analysis of different neuron types based on spike duration (new Figure 10, new Figure 11B, and Extended Data Figure 10-1). We have provided additional discussion of the physiology of MU vs. SU signals (page 35-36, lines 787-805) and fast vs. regular spiking neurons (page 40, lines 894-905 and new Figure 12B).

For the practical purpose of describing the signals in the MU activity, this may be okay.

MU activity is easy to record, and sometimes it is the only activity possible to record (e.g. if using fixed arrays as is often the case for BMI). The results, as presented, could be useful for an important but limited audience.

RESPONSE: We agree and have provided the additional requested analysis and discussion (see details below).

For the broader audience of neuroscientists, however, the manuscript falls short in its analyses. A more apples-to-apples comparison of MU and SU activity could be accomplished, we think, which would have more impact. This could involve constructing simulated MU activity by summing the signals of random subsets of SU neurons (using all 312 of them, not just the ones passing the inclusion criteria). Would that summation explain the results found for the MU activity, e.g. the loss of signal bimodality (Figure 7) or the smoother T-G transition (Figure 9)? If so, how many of the SU neurons need to be summed to achieve similarity with MU activity? That could give one an estimate of the actual numbers of neurons contributing to the MU samples. One could imagine a lot of analyses like this, based on unscreened SU neurons to simulate MU activity, that could be very illuminating to the field. It's a unique, beautiful MU and SU dataset, expertly collected from the same sites, monkeys, and tasks, that presents a valuable but missed opportunity for higher impact with just some further analyses.

RESPONSE: Thank you for this suggestion. In considering the best to reconstruct an MU population from spike-sorted SU data we arrived at the two most important factors:

1) our model fits assume identical behavioral conditions, so we would need to reconstruct and analyze MU response fields from SU data recorded and isolated in the same session. 2) the ideal 'apples to apples' comparison would be to compare these reconstructed response fields directly to site-matched original MU response fields recorded in the same sessions.

3 ACTIONS: As noted above, to create reconstructed MU response fields, we recombined all the activity from the SU (tuned, untuned, unresponsive, post-saccadic) from each of our MU sites (n = 103) to reconstruct the MU response field and repeated our best fit (Figure 9) and spatiotemporal analysis (Extended Data Figure 11-1) on these sites.

We then compared the results with the same analysis in the original MU activity. Figure 9 shows the distribution of spatial fits for the reconstructed MU data (Y axis) and original site-matched data fits (X axis) both for the visual responses (A; N = 88) and motor responses (B; N = 90). The scatter plots show reconstructed response field fits plotted as a function of original fits, matched for each recording site / session. As described in the text (Page 28-29, lines 617- 651), the reconstructed data fits still showed a mild tendency towards T for the visual response and G for the motor response, but they lost the clear unimodal distributions and peaks seen in our original multiunit data, becoming more spread out. Second, there was no significant correlation between the best fit scores for reconstructed MU plotted as a function of site-matched original MUs. This shows that the MU data cannot be explained simply by summation of the spike sorted SU data: something in the original dataset (which includes other noise and multiple baseline signals lost in spike sorting) seems to be important in providing a meaningful signal for MU data analysis. This is discussed on page 35-36 (lines 787-804).

2. Potential sample bias in SU signals vs. MU aggregate signals: Single-unit recording is well known to be affected by sample biases. As one example, neurons with larger extracellular potentials are easier to isolate, which means that larger pyramidal neurons may be over-represented (e.g. Towe &Harding 1970, Exp. Neurol. 29: 366-381).

Investigator biases can occur as well, such as focusing on fast firing rate neurons or neurons that seem especially task-related. Sampling biases may also affect MU recordings, although less is known about that. Potential sampling biases are not addressed at all in the manuscript, but it seems possible that they may contribute to the differences found between SU and MU samples.

RESPONSE/ACTIONS: This is a complex issue because there are many factors involved. We will try to break down possible sources of bias, and their likely effects, as follows: “ Experimental Conditions and Recording bias: To avoid this source of bias, MU and SU data were derived from the same dataset (pages 13, lines 282-282, line 289 and Extended Data Figure 11-1). “ 'Apples to Apples' comparisons: Further, in the scatter plots in Figure 8 (SU vs.

SU, MU vs. MU) and new Figure 9 (Reconstructed MU vs. Original MU), the sessions were identical and matched (pages 13, lines 282-282). “ Anatomic Distribution. Any factor that led to different anatomic distributions in the MU and SU data could create a bias. However, there was no correlation between anatomic sites and coding in our SU or MU data, and our sites largely overlapped, so this was unlikely to have an effect (Extended Data Figure 8-1). “ Spike Sorting: As noted above by the reviewers there is a bias in spike sorting toward 'big' neurons (neurons closer to the electrode, higher firing rates etc.), 4 excluding 'small' neurons, multiple baselines, and noise. This is the simplest explanation for the difference in the SU and MU data (page 12, lines 267-270). “ Quality of data: to ensure that the data were of good quality for our model fitting procedure we only analyzed data with significant spatial tuning (pages 17-18, lines 379-403). “ Exclusion Criteria: it is possible that removing data (such as untuned neurons) created a bias. Notably, the SU and MU sites were from the same sessions (page 13, lines 282-285, line 289 and Extended Data Figure 11-1). “ To see what affect untuned data would have on our populations, we created untuned and untuned + tuned datasets. In the spatiotemporal analysis (Extended Data Figure 11-1) we found that the untuned data added noise without affecting the overall visuomotor transformation (pages 32-33, lines 728- 735). However, when we examined the TG fit distributions of combined (tuned + untuned + unresponsive) SU visual and motor responses we found that this did not remove the cognitive signals, suggesting that untuned neurons do not cancel out the sensory and motor codes in the tuned population (pages 28-29, lines 617-651). “ Cortical depth / cell types: see response to next comment.

We also note that several papers have evaluated the putative types of neurons that can be recorded as SU activity in the FEF, based on action potential waveform shapes and other criteria (Ding &Gold 2012, Cerebral cortex, 22: 1052-1067; Shin &Sommer 2012, J Neurophysiol. 108: 2144-2159; Thiele et al. 2016, J Neurosci., 36: 7601-7612; Trainito et al. 2019, Current Biology, 29: 2973-2982; Dasilva et al. 2019, PNAS, 116: 20180- 20189). One might consider applying that sort of approach to evaluate whether the SU samples of this report seem biased toward pyramidal neurons or not. If a reasonably uniform distribution of putative pyramidal and inhibitory neurons is found, it could provide an argument that the SU samples are similar to the MU samples in terms of neural types. Or, if the SU sample seems biased toward one type of neuron or another, the investigators could leverage that information to adjust for sampling bias if they model the MU activity using the entire SU dataset as suggested in point 1 above.

RESPONSE: This is another potential sampling bias; we thank the reviewers for this suggestion. In the original paper we focused on Visual vs. Motor responses and did not subdivide these further. Although we recorded throughout the depth of FEF in each session, it is possible that sampling bias affected our population and SU dataset: regular spiking (RS) pyramidal neurons or fast spiking (FS) inhibitory neurons.

ACTION: Based on their physical properties, we separated our SU data into 69 RS and 51 FS neurons, which seems to be a reasonable distribution based on the literature cited above. We then repeated our analysis on each population separately (new Figures 10 and 11 B) and found an interesting result. Although FS and RS visual and motor codes were similar (Fig. 10), Figure 11B shows that FS neurons show a sudden shift toward gaze coding during the late delay period, compared with RS neurons. After 5 this sudden shift, the coding was mostly maintained in FS neurons until the gaze shift.

In contrast, RS neurons showed more a gradual transformation with several dips / rises in the code. Moreover, the FS coding was always closer to gaze than RS coding. This suggests that excitatory and inhibitory circuits seem to contribute complementary codes toward the population. We have provided a separate section in 'discussion' to discuss these results: Interactions between the regular-spiking (RS) and fast-spiking (FS) neurons (page 40, lines 894-905), updated our conceptual model to include these different neuron types (new Figure 12B), and cite the literature provided by the referees.

3. Bottom line: The main conclusion of the manuscript is that task-related signals in MU activity are more homogeneous than those in SU activity, but that is not particularly surprising. MU activity was screened for inclusion but must have contained many individual neurons that would fail the screening, and yet that MU activity was compared only to SU neurons that passed the screening. It is clear from the manuscript that the investigators are experts in modeling and neurophysiology. The manuscript would benefit from application of that expertise to a quantitative explanation of MU signal homogeneity (as suggested in points 1 and 2 above), or by a more scholarly discussion that carefully addresses the possible reasons for discrepancies in SU and MU signal content, or both. The Discussion Figure 10 and its associated text addressed the SU activity in depth, but the physiological factors that could explain the MU findings were not addressed with comparable clarity and detail.

RESPONSE/ACTION: Thank you, we believe that the additional analysis suggested by the reviewers adds nuances to the story without altering our original main conclusions.

Based on these data we have expanded our discussion of the differences between MU and SU population codes and possible reasons for those differences in the Discussion section (pages 35-36, lines 787-805).

Minor comments:

Lines 70-71: "Further, MU activity profiles are similar to local field potential (LFP) signals but tend to show less correlation between nearby neurons..." Do the authors mean "sites" instead of "neurons" at the end? Each MU activity profile is from a lot of neurons.

Or do the authors mean correlation between neurons extracted (sorted) from a single MU recording? Could be clarified.

REPLY/ACTION: We have made this clearer on page 4, line 77-79, as follows: Further, MU activity profiles are like local field potential (LFP) signals, but their activity tends to show less correlation with the activity of nearby neurons.

Line 147: Possible confusing typo, maybe "coils" instead of "cells"? ACTION: This is corrected to 'coils' (page 8, line 175) 6 Lines 241-243 and elsewhere: "Figure 2C-D displays the overlapped and averaged spike density plots for top 10 % (for each neuron and MU site) trials for the SU and MU." We found it confusing why 90% of trials were omitted from analysis, until we realized that this is how to isolate trials from the hot spot of the receptive or movement field given the mapping techniques used. It will help the readers a lot if the authors explain the criteria and rationale for picking the top 10% of trials, particularly since the manuscript should not assume that readers are familiar with previous papers with respect to this fundamental step in the analyses.52-253 and page 12, lines 256-257 ACTION: This has been further clarified in the manuscript in the caption to Figure 2. We used all trials to do our response field fits (page 50, lines 1345-1346).

Lines 269-270: "As the MU cluster comprises different spikes, it generally has a lower amplitude than the individual spikes sorted from it." This seems like an overly simplistic, and potentially misleading, explanation. It's more that the electrode isn't immediately near any one neuron, so one collects more signals, but they are all further away than for an SU signal (assuming the same density of neurons for MU and SU recordings). We suggest rewording this explanation, particularly explaining the following: what do you mean by cluster, is it the nearby tissue at the recording site? When the authors write amplitude do they mean the resulting amplitude after applying the single unit threshold to the multi-unit data? Please reformulate this sentence for clarity.

ACTION: This has been rephrased accordingly on pages 12-13 (lines 258-280).

Lines 270-272: "We re-thresholded the high-pass filtered raw data to isolate the MU activity from the 40 kHz signal with a spike detection threshold of 3.5 standard deviations (SDs). What 40 kHz signal? We don't see that explained anywhere. And 3.5 SDs of what? The noise level? For replication and reproducibility, this method should be explained better.

REPLY: We recorded the neural signal at 40 KHz sampling rate after two amplification steps. The MU activity was isolated from the raw signal with a spike detection threshold of 3.5 standard deviations above the mean noise level.

ACTION: This section has now been rewritten with more details on neural recordings (Pages 12-13, lines 258-280).

Line 273: "[the procedure described in point 5 above] led us to spiking activity recorded from several neurons recorded within a vicinity of ∼100-200 μm around the electrode tip." It seems odd to state that estimated radius so matter-of-factly. Perhaps this could be qualified a bit, perhaps like "According to previous studies, the neurons were likely ~100-200 um from the electrode tip, compared to ~X um from single neurons in the SU recordings" (and fill in the X, it would be useful for readers to understand that comparison).

7 ACTION: We have rephrased it accordingly (Page 12-13, lines 275-280).

Figure 3 legend: L and L' abbreviations are defined but not used in the figure anywhere we could see.

ACTION: We have now added L and L' in the figure.

Line 336: "Sum" of Squares, not "Some." ACTION: We have corrected this (Page 15, Lines 326).

Lines 370-372 and elsewhere: "The spatial continuum between Te and Ge ('T-G continuum') provided the best overall description of the data..." Here, and later in the paper up until Figure 7, the continuum is described as ranging from Te to Ge. But in Figure 7 many values are shown Ge. This is a confusing disconnect between the text and that Figure. Please clarify the text in a way that readers aren't surprised by the range of values displayed in Figure 7.

RESPONSE/ACTION: We have clarified that the fits sometimes go beyond the T-G range, so to avoid edge effects 10 steps are included 10 more steps on either side of T and G (page 17, line 371).

Line 395: "Thus, we removed the time steps..." Please provide a quantification on how many time steps were removed, on average, per session.

ACTION: We have now added plots (Figure 11, Extended Data Figure 10-1C, and Extended Data Figure 11-1) for the spatiotemporal analysis at the bottom of all the figures to show the percentage of spatially tuned neurons at each time step.

Lines 398-400: "CI for an SU / MU site, ..." is not a complete sentence.

ACTION: We have corrected this (Page 18, Lines 397-398).

Figure 4B and 4D: The p-value for dE was also very close to 1, it appears. Numerically, Ge wins, but it seems odd to not even mention that dE is nearly equally well fitted.

ACTION: We have now added that in the text (Page 21, lines 471-472).

Line 406-407: "To accommodate this variable trial-time for transformations between these events, we time-normalized the duration of each trial." It would help the reader if that sentence were followed with something like, "This normalization was conducted 8 only for the Spatiotemporal Analysis presented at the end of Results." We were confused throughout the Results, expecting to see the normalizations, until we got to the final section where it was used for Figure 9. Please rewrite.

ACTION: We have now added a sentence following the above sentence (Pages 18-19, lines 410-412).

Lines 498 and 531: Would help the reader to reword these opening sentences as "shows the visual response field" and "shows the motor response field," respectively.

ACTION: We have added that in both cases (page 22, line 495 and page 23, line 517).

Line 567: "...peaked in different quadrants upper-left......upper-right...." Should read: peaked in different quadrants lower-left......upper-left.

ACTION: We have added the quadrant numbers Figure 5 and also corrected the text (Page 23, line 520).

Line 569: "(the second neuron, not shown, ...)". Do the authors mean "a" second neuron? We don't see anywhere else that it was mentioned that two SUs were isolated from this recording.

ACTION: We have now mentioned in the text that two neurons were recorded from the site (Page 23, lines 512-513).

Line 570: The authors write: ... second quadrant. The authors never defined a ranking order for quadrants (first vs second etc..). Please clarify.

ACTION: We have now indicated that on the first instance when the example response field is presented (Figure 5 C).

Line 627: "one sampled Wilcoxon signed rank test" - against what theoretical value? ACTION: We have provided the theoretical value in the text now (Page 24, line 539).

Line 628: Fig. 8B2 should read: 7B2.

ACTION: We have corrected this (page 25, line 561).

Line 712: "... much smoother..." Please provide a quantification of this effect. One might just compare the sum of the unsigned differences between 2 successive data points.

9 ACTION: We have performed a statistical test between the corresponding data points (page 32, lines 712-719). We rearranged the text to report both quantitative and qualitative aspects. Followed by the statistical test, we mention that though there was no statistical difference between the curves, there 'appears to be a smoother' transformation in MU than SU. ...............................

Line 870: Figure 7C, must read Figure 6C.

ACTION: We have corrected this (Page 41, line 927)
