# Supplementary figures and images for: Multiunit Frontal Eye Field Activity Codes the Visuomotor Transformation, But Not Gaze Prediction or Retrospective Target Memory, in a Delayed Saccade Task
Source: eNeuro. 2024 Aug 6;11(8):ENEURO.0413-23.2024. doi: 10.1523/ENEURO.0413-23.2024 (PMC11373882; doi:10.1523/ENEURO.0413-23.2024)

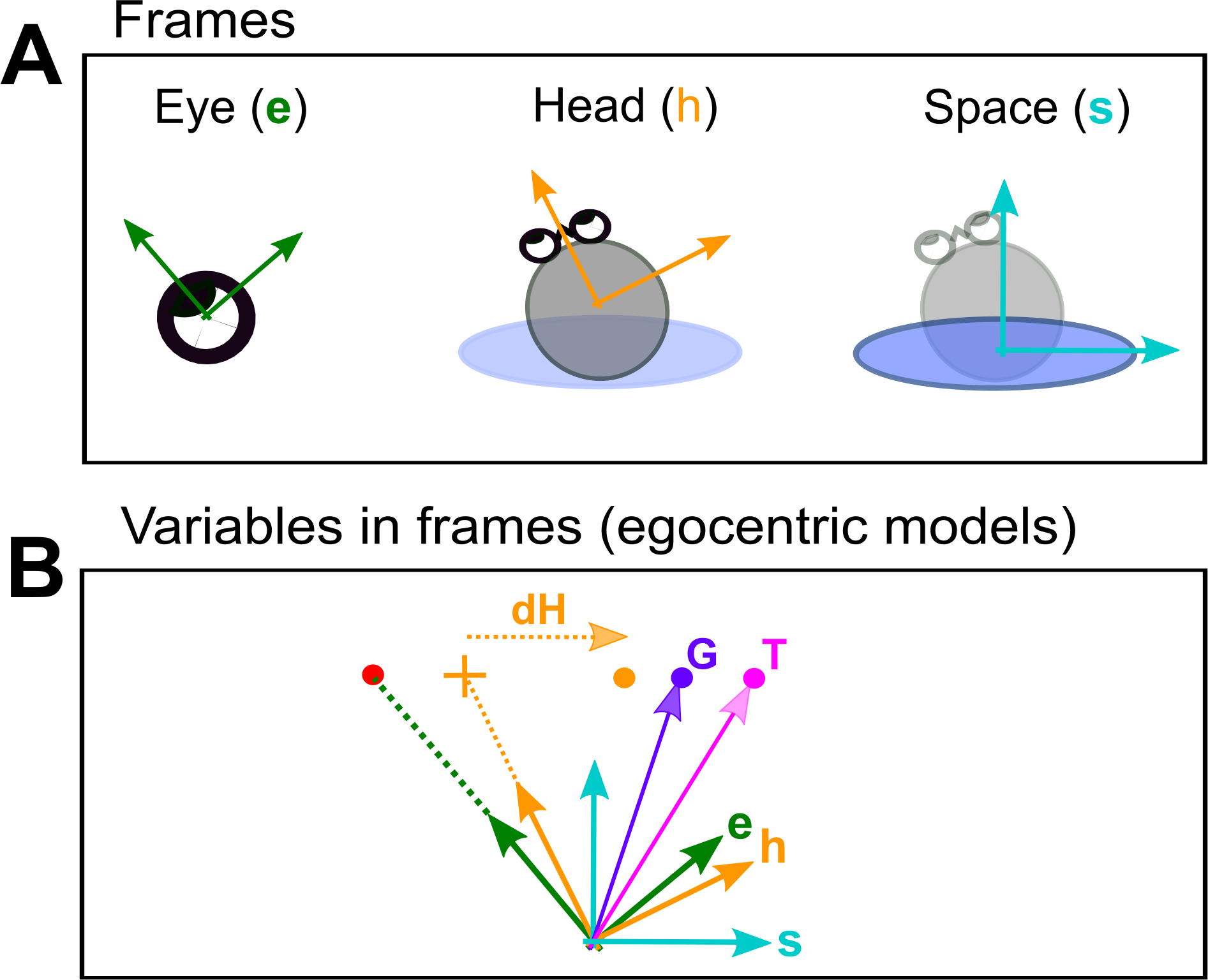

Supplement: Figure 3-1 — A, Basic egocentric reference frames: eye (e), head (h) and body / space (s). B, Different variables, and egocentric models for an example trial. Models tested: the difference between the initial and the final head orientation relative to space (dH); the difference between the initial and the final eye orientation relative to the head (dE); future orientation of the head in space coordinates (Hs); Eye in head (Eh); Future gaze in space (Gs); Future gaze in head (Gh); Future gaze in eye (Ge); Target in space (Ts); Target in head (Th); Target in eye (Te). Download Figure 3-1, TIF file. [file eneuro-11-ENEURO.0413-23.2024-s001.tif]

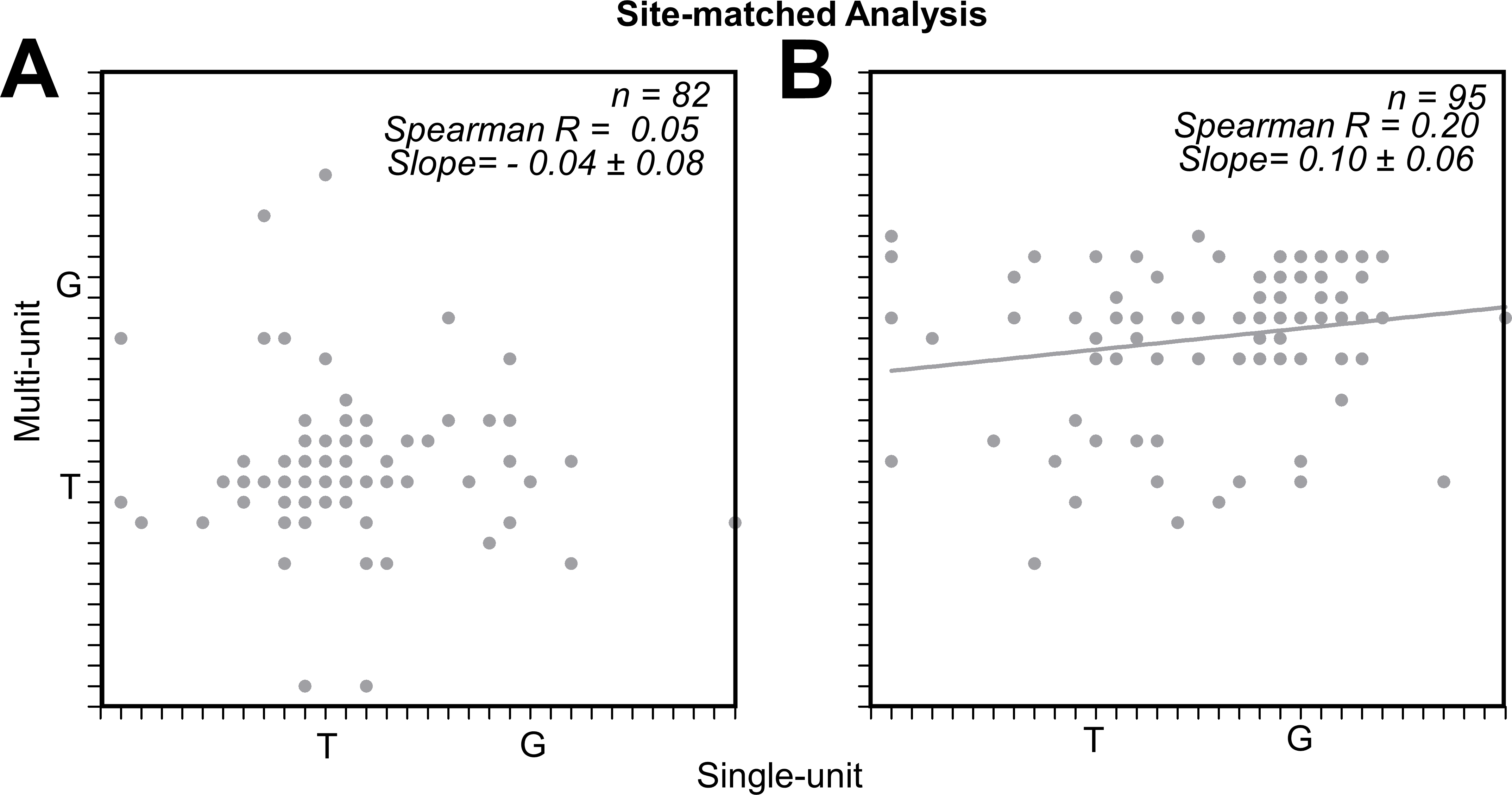

Supplement: Figure 8-1 — Site-matched analysis. A, Visual MU sites as a function of the corresponding best fit score of visual SUs. No significant correlation was observed (Spearman R = 0.05, Slope = - 0.04 ± 0.08, p = 0.66). B, Motor MU sites as a function of the corresponding best fit score of motor SUs. A modest correlation (Spearman R = 0.20, Slope = 0.10 ± 0.06) but nearly reaching significance was observed (p = 0.053). Download Figure 8-1, TIF file. [file eneuro-11-ENEURO.0413-23.2024-s002.tif]

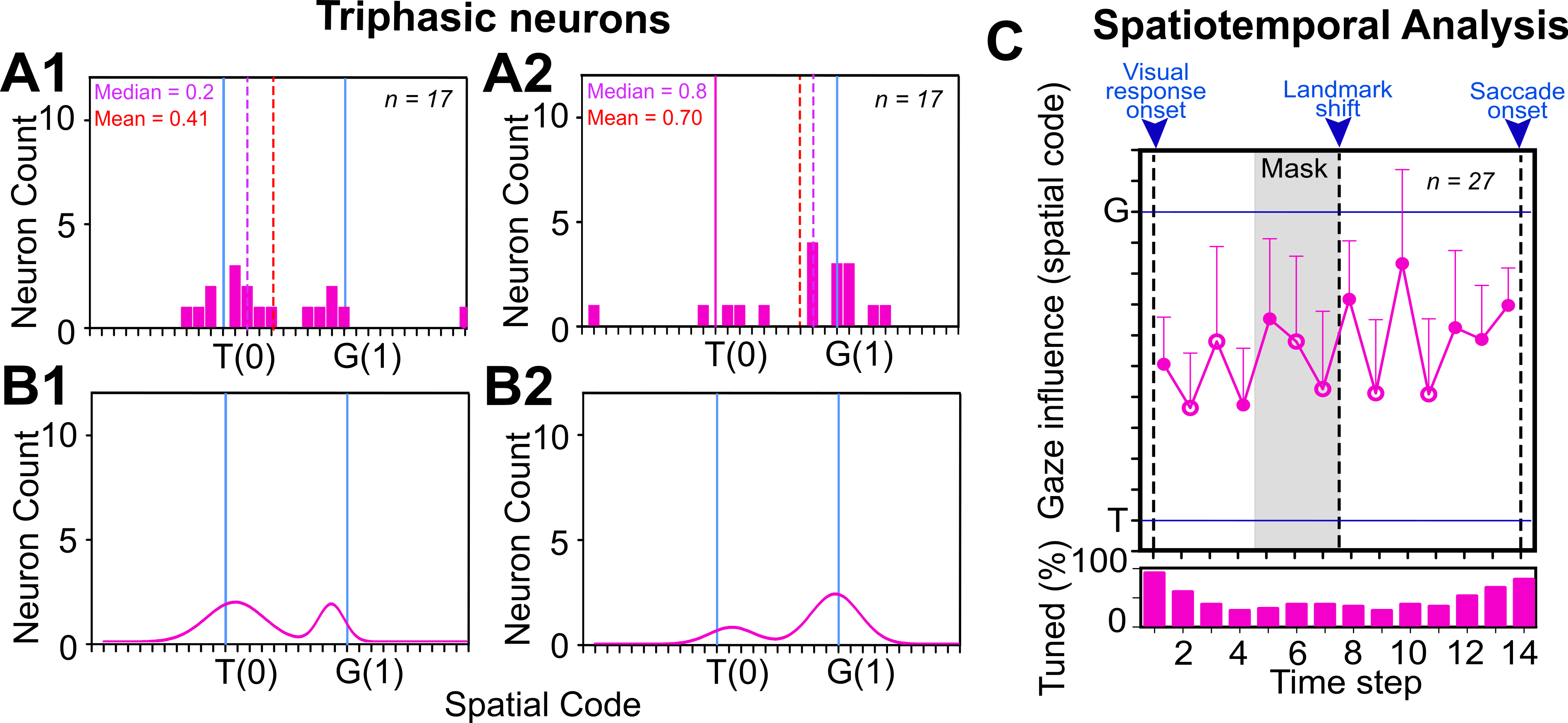

Supplement: Figure 10-1 — Triphasic waveforms. A1, Frequency distribution of best fits of spatially tuned visual responses (n = 17, mean = 0.41; median = 0.2), significantly shifted (p = 0.011, one-sampled Wilcoxon signed rank test). A2, Frequency distribution of best fits of spatially tuned motor responses (n = 17, mean = 0.7; median = 0.8), significantly shifted from T toward G (p = 0.0003, one-sampled Wilcoxon signed rank test). B1-B2, The sum of two Gaussian distributions for visual (B1) and motor (B2) responses (bimodal distribution in both cases). C, Spatiotemporal progression for triphasic neurons (n = 27), characterized by several dips and rises from visual to motor responses. The solid circle represents a significant shift (p < 0.05, one-sampled Wilcoxon signed rank test) from T toward G. Download Figure 10-1, TIF file. [file eneuro-11-ENEURO.0413-23.2024-s003.tif]

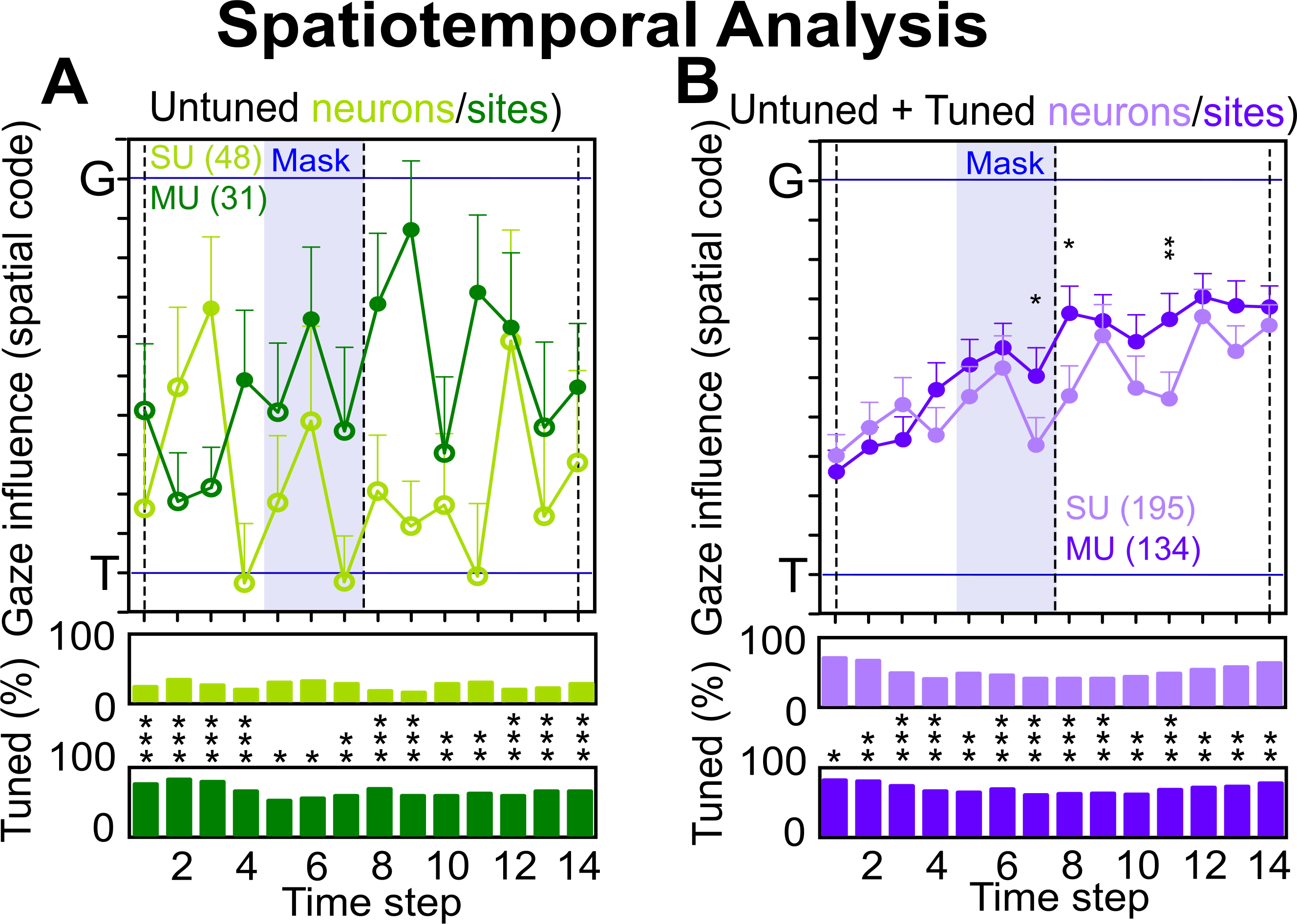

Supplement: Figure 11-1 — A, Spatiotemporal progression for untuned neurons / sites (n = 48 / 31). No clear trend was observed for the SUs (light green) but the corresponding sites (dark green) showed a weak signature of T-G progression. B, Spatiotemporal progression for tuned + untuned neurons / sites (n = 134 / 195). A similar progression to the tuned only neurons / sites (Fig. 10), but untuned neurons seem to dilute the TG progression (significant difference between SU and MU at time steps 7, 8, and 11; p < 0.05, Mann-Whitney U test). The solid circle represents a significant shift (p < 0.05, one-sampled Wilcoxon signed rank test) from T toward G. At all steps (A and B), the corresponding proportions of spatially tuned data were significantly different from each other. Download Figure 11-1, TIF file. [file eneuro-11-ENEURO.0413-23.2024-s004.tif]
